# A critical role for STAT3 Thr714 phosphorylation in NPM-ALK-driven tumorigenesis

**DOI:** 10.1038/s41598-026-44867-w

**Published:** 2026-03-25

**Authors:** Xin Lin, Yoshiyuki Yao, Yasuhiro Moriwaki, Kenji Tago, Megumi Funakoshi-Tago

**Affiliations:** 1 Division of Hygienic Chemistry, Faculty of Pharmacy, Keio University, 1-5-30 Shibakoen, Minato-ku, Tokyo, 105-8512 Japan; 2 Education Research Center for Pharmaceutical Sciences, Faculty of Pharmacy, Keio University, 1-5-30 Shibakoen, Minato-ku, Tokyo, 105-8512 Japan; 3 Department of Laboratory Sciences, Gunma University Graduate School of Health Sciences, 3-39-22 Showa-Machi, Maebashi, 371-8514 Gunma Japan

**Keywords:** NPM-ALK, STAT3, Phosphorylation, T714, Tumorigenesis, Cancer, Cell biology, Molecular biology

## Abstract

The oncogenic fusion protein NPM-ALK drives anaplastic large cell lymphoma (ALCL) by activating the transcription factor STAT3. While STAT3 phosphorylation at Y705 and S727 is well characterized, the present study defines a mechanistic role for phosphorylation at T714 in supporting full STAT3 functionality. In NPM-ALK–positive ALCL cells, STAT3 is phosphorylated at Y705, S727, and T714, and this is suppressed by ALK inhibition. Enforced NPM-ALK expression in Ba/F3 cells induces phosphorylation at all three sites in a kinase-dependent manner. To investigate the role of T714, wild-type STAT3 or a T714A mutant was reconstituted into STAT3-knockdown Ba/F3 cells expressing NPM-ALK. Wild-type STAT3 underwent Y705 and S727 phosphorylation and nuclear translocation, whereas the T714A mutant was phosphorylated at S727 only and failed to translocate. The reduced expression of STAT3 target genes (Cyclin D1, Pim1, Pim2, and Socs3) with STAT3 knockdown was restored by wild-type STAT3, but not by the T714A mutant. In vivo, STAT3 knockdown suppressed tumor formation and hepatosplenomegaly in mice inoculated with Ba/F3 cells expressing NPM-ALK, and these phenotypes were rescued by wild-type STAT3, but not by the T714A mutant. These findings indicate that STAT3 phosphorylation at T714 is required for subsequent Y705 phosphorylation, nuclear translocation, and transcriptional activation specifically within the context of NPM-ALK–mediated signaling.

**Supplementary Information:**

The online version contains supplementary material available at 10.1038/s41598-026-44867-w.

## Introduction

Anaplastic large cell lymphoma (ALCL) is a subtype of T-cell lymphoma characterized by the elevated expression of the surface marker CD30^1^. A subset of ALCL cases carries the chromosomal translocation t(2;5)(p23;q35), which generates the oncogenic fusion protein NPM-ALK^2^. This fusion protein combines the oligomerization domain of nucleophosmin 1 (NPM1)—a multifunctional nuclear protein—with the kinase domain of anaplastic lymphoma kinase (ALK), a receptor tyrosine kinase belonging to the insulin receptor superfamily^3,4^. The resulting NPM-ALK forms constitutively active homodimers via the NPM1-derived oligomerization domain, leading to persistent autophosphorylation and the activation of downstream signaling pathways^5^. NPM-ALK activates multiple pro-survival signaling pathways, including those mediated by signal transducer and activator of transcription 3 (STAT3), thereby contributing to malignant transformation^6–8^. Functional analyses demonstrated that the genetic ablation of Stat3 impaired NPM-ALK–dependent transformation in murine fibroblasts and suppressed lymphomagenesis in transgenic models, underscoring the critical role of STAT3 activation in NPM-ALK–driven oncogenesis^9^.

STAT3 consists of multiple functional domains, including the N-terminal domain, coiled-coil domain, DNA-binding domain, Src homology 2 (SH2) domain, and transactivation domain^10,11^. Its transcriptional activity is modulated by phosphorylation at several residues within the C-terminal region, notably Y705, T714, and S727^12–14^.

Phosphorylation at Y705, typically mediated by tyrosine kinases, such as the JAK and Src families, enables STAT3 to form homodimers through reciprocal SH2 domain interactions^15,16^. These dimers translocate to the nucleus, recognize specific DNA motifs (e.g., TTCC[G/C]GGAA), and initiate the transcription of target genes^17^. In contrast, S727 phosphorylation, often catalyzed by MAP kinase family members, has been implicated in diverse cellular outcomes^18,19^. It may enhance STAT3’s transcriptional potency or facilitate its mitochondrial localization, thereby affecting mitochondrial function and metabolism^20–22^. More recently, T714 phosphorylation has emerged as a regulatory event that is mediated by GSK-3α/β in endothelial cells upon the co-activation of epidermal growth factor receptor (EGFR) and protease-activated receptor-1 (PAR-1). This modification was shown to contribute to STAT3-dependent gene induction in that context^14^.Collectively, these findings suggest that STAT3 activity is governed by a multilayered phosphorylation code, with each site exerting distinct regulatory functions depending on the cellular context and upstream signaling inputs.

We previously reported that phosphorylation at Y705 was essential for the NPM-ALK–mediated expression of STAT3 target genes and cell proliferation, whereas phosphorylation at S727—induced via the NPM-ALK–activated JNK pathway—contributed to STAT3 protein stabilization^23^. However, it currently remains unclear whether STAT3 is phosphorylated at T714 downstream of NPM-ALK in ALCL cells, and also if this modification plays a functional role in NPM-ALK–driven transformation. The present study demonstrated for the first time that NPM-ALK induced STAT3 phosphorylation at T714, in addition to Y705 and S727, in a kinase activity–dependent manner. Furthermore, using STAT3-knockdown Ba/F3 cells expressing NPM-ALK and reconstituted with the STAT3 T714A mutant, we showed that phosphorylation at T714 was indispensable for NPM-ALK–mediated cellular transformation, thereby establishing its functional significance in this oncogenic pathway.

## Materials and methods

### Reagents

Crizotinib (PF-02341066) was kindly provided by Pfizer (San Diego, CA, USA). Alectinib was purchased from LC Laboratories (Woburn, MA, USA). Recombinant murine IL-3 was obtained from PEPROTECH (Rocky Hill, NJ, USA), and puromycin and G418 were purchased from InVivoGen (San Diego, CA, USA). A polyclonal antiserum specific for phospho-STAT3 (T714) was generated by immunizing rabbits with a synthetic phosphopeptide (KFICVpTPTTC) corresponding to the T714 phosphorylation site, following previously described protocols^14^. Antibodies against phospho-ALK (Y1604), total ALK, phospho-STAT3 (Y705), phospho-STAT3 (S727), total STAT3, MEK1/2, and HRP-conjugated secondary antibodies (rabbit anti-mouse and goat anti-rabbit) were obtained from Cell Signaling Technology (Danvers, MA, USA). Antibodies against β-actin and Lamin B were purchased from Santa Cruz Biotechnology Inc. (Santa Cruz, CA, USA).

### Plasmids

The cDNA encoding NPM-ALK was inserted into the MSCV-IRES-GFP retroviral vector^8^. pLEGFP-WT-STAT3 was a gift from George Stark (Addgene plasmid # 71450 ; http://n2t.net/addgene:71450 ; RRID: Addgene_71450). The threonine-to-alanine substitution at residue 714 of STAT3 (T714A) was generated using a site-directed mutagenesis kit (Stratagene, CA). As previously reported^23,24^, the oligonucleotide sequences used to construct the shRNA-expressing retroviral vector targeting STAT3 were as follows: 5′- gatcccc*gcatcaatcctgtggtata*ttcaagaga*tataccacaggattgatgc*ttttta-3′ and 5′-agcttaaaaa*gcatcaatcctgtggtata*tctcttgaa*tattgctgcaggtcgttgg*ggg. Underlined sequences correspond to the sequence of murine STAT3 (from 1491 to 1509 in ORF). The annealed oligonucleotides were inserted into pSuper-retro-puro. The nucleotide sequences of wild-type STAT3 and the STAT3 T714A mutant were substituted into the nucleotide sequences resistant to sh-STAT3 by mutagenesis PCR.

### Cell culture and retrovirus infection

The murine pro-B cell line Ba/F3 was obtained from the RIKEN BioResource Center (Tsukuba, Japan). SUDH-L1 cells and Ki-JK cells, both derived from NPM-ALK-positive ALCL patients, were purchased from the American Type Culture Collection (Manassas, VA, USA) and the JCRB Cell Bank (Osaka, Japan), respectively. These cells were maintained in RPMI-1640 medium (Nacalai Tesque, Kyoto, Japan) supplemented with 10% fetal bovine serum (FBS) (BioWest, Nuaillé, France), 100 U/mL penicillin, 100 µg/mL streptomycin (both from Nacalai Tesque), and 2 ng/mL recombinant murine IL-3 (PEPROTECH).

The retroviral transduction of Ba/F3 cells was conducted using RetroNectin-coated plates (Takara Bio Inc., Shiga, Japan), following previously established protocols^8^. After infection, cells were selected in medium containing 5 µg/mL puromycin and 8 µg/mL G418 (InVivoGen, San Diego, CA, USA).

### Immunoblotting

Whole-cell lysates were prepared using NP-40 lysis buffer containing 50 mM Tris-HCl (pH 8.0), 120 mM NaCl, 1 mM EDTA, 0.5% Nonidet P-40, 20 mM NaF, 0.2 mM Na3VO4, 2 µg/mL aprotinin, and 2 µg/mL leupeptin. Cytoplasmic and nuclear fractions were separated by sequential lysis using hypotonic and detergent-containing buffers following established procedures^25^. SDS-denatured protein samples from whole-cell lysates and subcellular fractions were separated by SDS-PAGE and transferred to PVDF membranes. To allow probing with different antibodies, the membranes were cut prior to antibody incubation. All original blot images, including all replicates, have been provided in the Supplementary Information. Band intensities were quantified using ImageJ software, and the relative levels of phosphorylated STAT3 and NPM-ALK were presented in graphical format.

### Assessment of phospho-STAT3 (T714) antibody specificity

To assess the specificity of the pT714 antibody, the antibody was used either alone or pre-incubated with 20 µg/mL of a phosphorylated STAT3 T714 peptide (KFICVpTPTTC)　or a non-phosphorylated STAT3 T714 peptide (KFICVTPTTC) at room temperature for 1 h. These antibody preparations were subsequently applied to immunoblotting using lysates derived from Ki-JK cells, SUDHL-1 cells, and NPM-ALK-expressing Ba/F3 cells.

For phosphatase treatment, cells were lysed using either a lysis buffer containing 50 mM Tris-HCl (pH 7.5), 150 mM NaCl, 1 mM MgCl₂, 1% NP-40, 2 µg/mL aprotinin, and 2 µg/mL leupeptin, or the same buffer supplemented with 400 U/mL of λ-phosphatase (New England Biolabs, Ipswich, MA, USA). The resulting lysates were subjected to immunoblotting using phospho-specific antibodies against STAT3 pT714, pY705, and pS727.

### Reverse transcription-polymerase chain reaction (RT-PCR)

RNA was prepared using Sepasol (Nacalai Tesque) and RT was performed using the Oligo (dT)_20_ primer and ReverTra Ace (TOYOBO, Tokyo, Japan) as previously described^23,24^. Real-time PCR was conducted using Luna Universal qPCR Master Mix (NEW ENGLAND Biolabs, MA, USA). The primer sequences used in the quantitative PCR analysis were as follows:

STAT3 (mouse) Forward:5’-CAAGGGCTTCTCCTTCTGGG-3’ Reverse:5’-GGGGGCTTTGTGCTTAGGAT-3’.

Cyclin D1 (mouse) Forward: 5′-TCCCAGACGTTCAGAACC-3′ Reverse: 5′-AGGGCATCTGTAAATACACT-3′.

Pim1 (mouse) Forward: 5′-CTTCGGCTCGGTCTACTCTG-3′ Reverse: 5′-CCGAGCTCACCTTCTTCAAC-3′.

Pim2 (mouse) Forward: 5′-AGTTGCCTTCTTGGGACTGA-3′ Reverse: 5′-TCCACGATTTCCCAGAGAAC-3′.

Socs3 (mouse) Forward: 5′-AGCTCCAAAAGCGAGTACCA-3′ Reverse: 5′-TGACGCTCAACGTGAAGAAG-3′.

β2-Microglobulin (mouse) Forward: 5′-CTGACCGGCCTGTATGCTAT-3′ Reverse: 5′-TCACATGTCTCGATCCCAGT-3′.

### Water-soluble tetrazolium (WST) assay and trypan blue staining

Ba/F3 cells (5 × 10^4^/well) were seeded on 96-well plates and cultured for 72 h. Cell viability was assessed using Cell Count Reagent SF (Nacalai Tesque) according to the manufacturer’s protocol, and absorbance at 450/690 nm was measured with Infinite 200 PRO (Tecan, Switzerland). Regarding trypan blue exclusion, cells (1 × 10^5^/well) were cultured in 48-well plates for 72 h, and viable cells were quantified using Vi-CELL (Beckman Coulter) as previously described^23^.

### Analysis of the cell cycle

Cells were fixed in 70% ethanol at − 20 °C overnight and were then resuspended in PBS containing 10 µg/mL RNase A (Nacalai Tesque). Propidium iodide (100 µg/mL) (Wako Pure Chemical Industries, Tokyo, Japan) was added to stain cellular DNA. Cell cycle profiles were analyzed by flow cytometry using FACSLSRII (BD Biosciences, San Jose, CA, USA), as previously described^24^.

### Tumorigenesis analysis using nude mice

Four-week-old female BALB/cSIc-nu/nu nude mice (Sankyo Lab Service Co., Ltd.) weighing approximately 15–18 g were used for transplantation experiments. Transduced Ba/F3 cells were resuspended in PBS at a concentration of 5 × 10⁶ cells per 200 µL and injected subcutaneously into the dorsal flank using a 1-mL syringe fitted with a 23G needle (Terumo Corporation, Tokyo, Japan). Starting on day 8 post-injection, tumor dimensions (length and width) were measured daily using Vernier calipers, and tumor volume was calculated using the following formula: volume = ½ × (length × width²), as previously described^26^. Tumor diameters (in cm) for each mouse are presented in Table S1. On day 18, mice (*n* = 6 per group) were anesthetized with isoflurane (3–5% induction; FUJIFILM Wako Pure Chemical Corporation, Osaka, Japan) until a surgical plane of anesthesia was reached, after which cervical dislocation was performed by trained personnel. Death was confirmed by the absence of respiration, heartbeat, and reflexes, and carcasses were disposed of in accordance with institutional biosafety regulations. Individual tumor, liver, and spleen weights for each mouse are provided in Supplementary Table S1. All animal experiments were conducted in accordance with institutional guidelines and approved by the Animal Usage Committee of Keio University (Approval No. A2022-298). This study was conducted and reported in accordance with the ARRIVE guidelines (https://arriveguidelines.org).

### Statistical analysis

All statistical analyses were performed using GraphPad Prism software (version 9, GraphPad Software, San Diego, CA, USA). Data are presented as mean ± standard deviation (SD). For comparisons between two groups, unpaired two-tailed Student’s *t*-tests were used. For comparisons among more than two groups, one-way analysis of variance (ANOVA) followed by Tukey’s multiple comparison test was applied. A *p*-value of < 0.05 was considered statistically significant.

In animal experiments, mice were randomly assigned to each group. Although blinding was not performed, data analysis was conducted objectively based on predefined criteria. The sample size (*n* = 6 per group) was determined based on previous studies using similar models and was considered sufficient to detect statistically meaningful differences in our experimental system.

## Results

### NPM-ALK induces the phosphorylation of STAT3 at Y705, T714, and S727 in a kinase activity–dependent manner

**Fig. 1:**
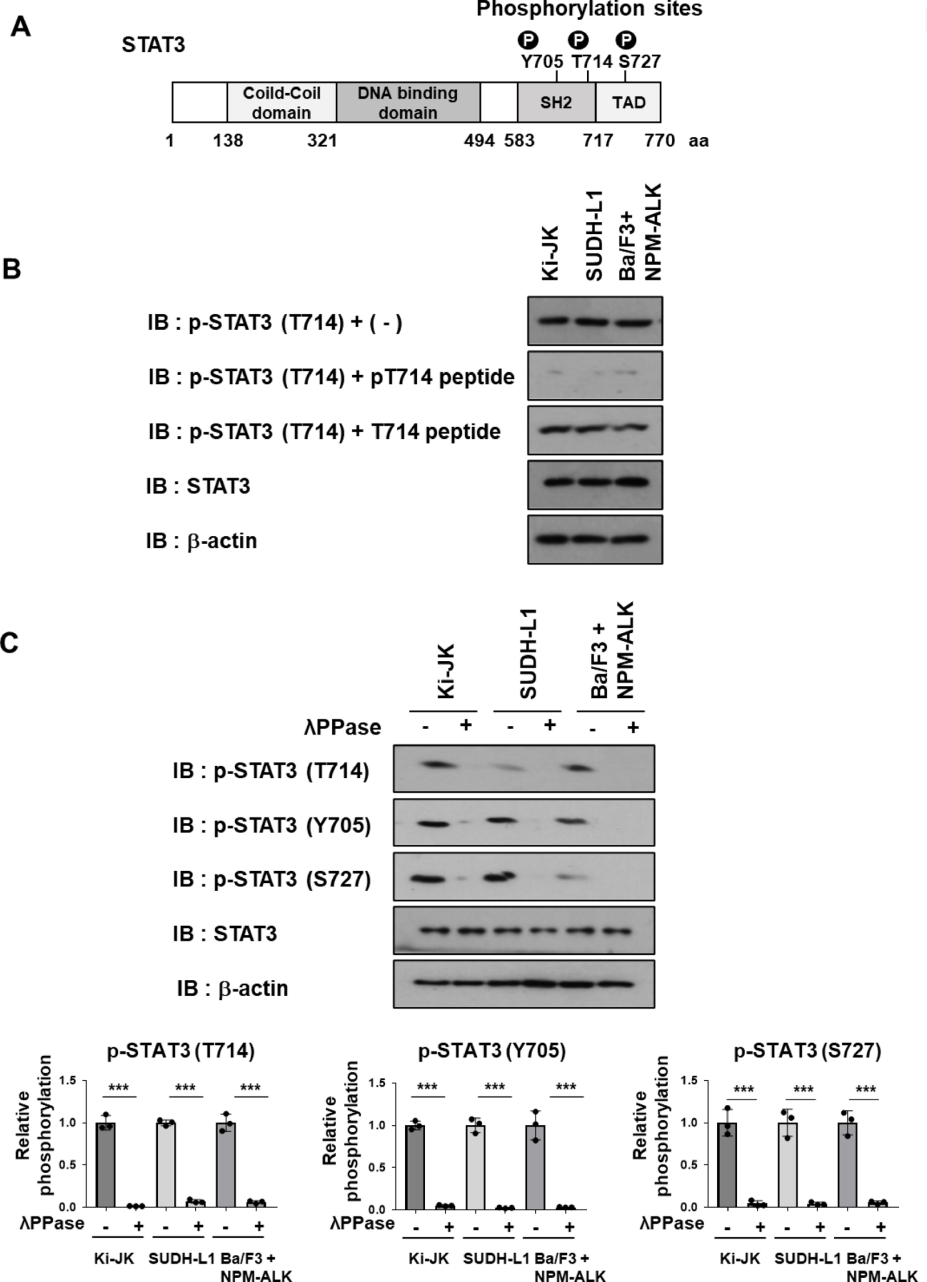
The anti-phospho-STAT3 (T714) antibody specifically recognizes STAT3 phosphorylated at T714. (**A**) Schematic representation of the STAT3 protein structure. The three phosphorylation sites—Y705, T714, and S727—are located in the C-terminal region. (**B**) Cell lysates were prepared from Ki-JK cells, SUDHL-1 cells, and Ba/F3 cells expressing NPM–ALK. Immunoblotting was performed using the anti-phospho-STAT3 (T714) antibody alone, or after pre-incubation with a phosphorylated STAT3 T714 peptide (pT714) or a non-phosphorylated T714 peptide (T714). Anti-STAT3 and anti–β-actin antibodies were used as controls. The membranes were cut prior to antibody incubation, and all original blot images are provided in the Supplementary Information. (**C**) Cell lysates were prepared using lysis buffer with or without λ-phosphatase (λPPase). Immunoblotting was performed using phospho-specific antibodies against STAT3 pT714, pY705, and pS727, as well as anti-STAT3 and anti–β-actin antibodies. The membranes were cut prior to antibody incubation. STAT3 phosphorylation levels were quantified (*n* = 3). The significance of differences was set as ***p < 0.001. All original blot images, including all replicates, are provided in the Supplementary Information.

Y705, T714, and S727, located within the C-terminal region of STAT3, are established phosphorylation sites (Fig. 1A)^12–14^. To assess the phosphorylation status of these residues, we used commercially available phospho-specific antibodies against Y705 and S727. However, since no antibody was available for phosphorylated T714, we generated a phospho-specific antibody against T714 using a synthetic phosphopeptide as the immunogen. To validate the specificity of this antibody, we first performed peptide competition assays using lysates derived from Ba/F3 cells expressing NPM–ALK and the NPM–ALK–positive ALCL patient-derived cell lines Ki-JK and SUDHL-1 A clear signal was detected in all cell lysates using the anti-phospho-STAT3 (T714) antibody. Peptide competition assays demonstrated that this signal was completely abolished by pre-incubation with the phosphorylated peptide (pT714), whereas no change was observed following incubation with the non-phosphorylated peptide (T714) (Fig. 1B). Furthermore, treatment with λ-phosphatase (λPPase) resulted in a marked reduction of the pT714 signal in all tested cell lines. Similarly, the pY705 and pS727 signals were also reduced following treatment, indicating that the signals detected by these phospho-specific antibodies are phosphorylation-dependent (Fig. 1C). These results suggest that the custom-made anti-phospho-STAT3 (T714) antibody specifically recognizes STAT3 phosphorylated at T714.

**Fig. 2:**
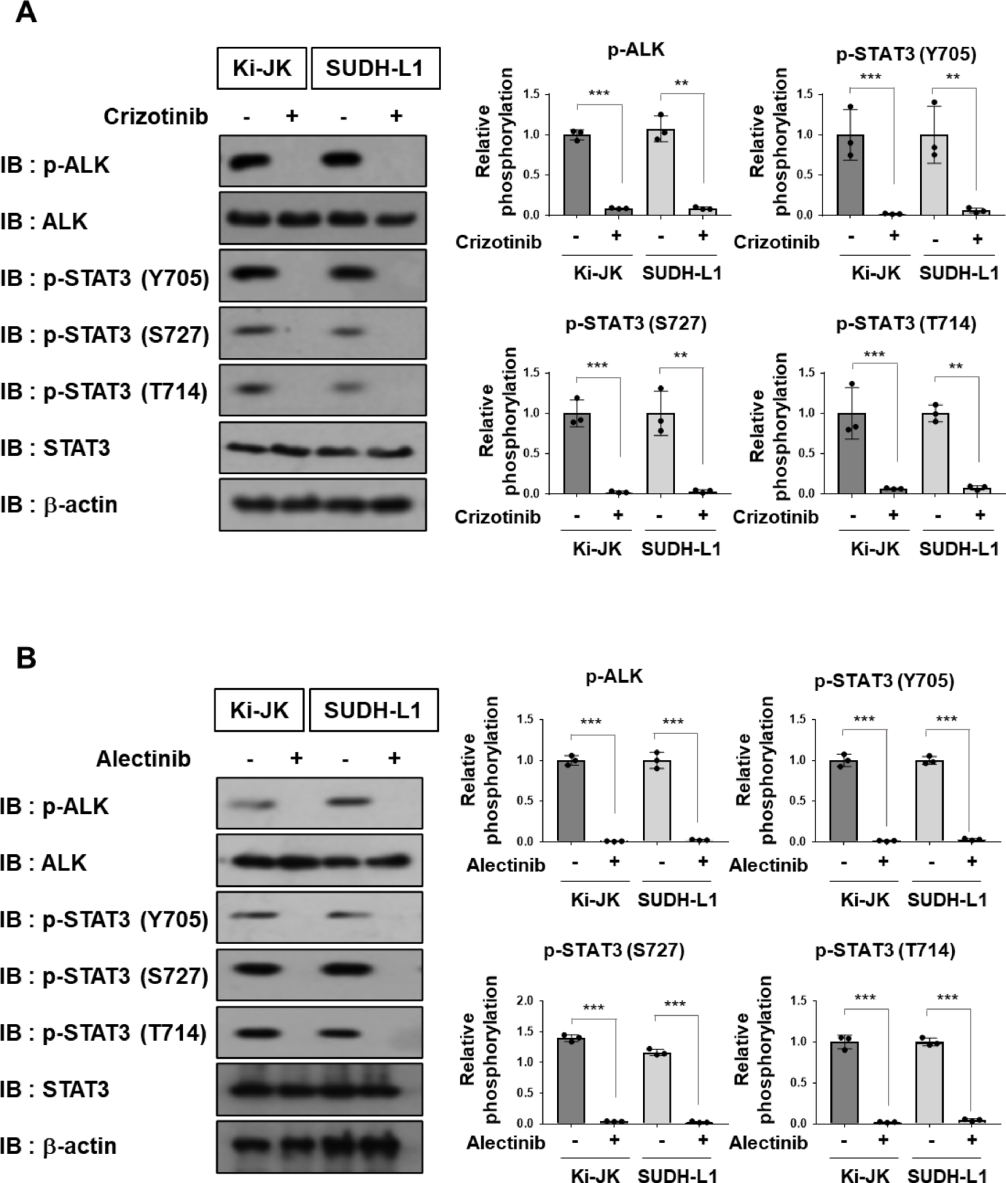
NPM-ALK induces STAT3 phosphorylation at Y705, T714, and S727 in a kinase activity-dependent manner in ALCL patient-derived cells. ALCL-derived Ki-JK and SUDH-L1 cells were treated with (**A**) 1 µM crizotinib or (**B**) 0.5 µM alectinib for 24 h. Whole-cell lysates were prepared and subjected to an immunoblot analysis. The membranes were cut prior to antibody incubation. ALK and STAT3 phosphorylation levels were quantified (*n* = 3). The significance of differences was set as **p < 0.01, ***p < 0.001. All original blot images, including all replicates, are provided in the Supplementary Information.

We next examined the effects of the first-generation ALK inhibitor crizotinib and the second-generation inhibitor alectinib on the three STAT3 phosphorylation sites (Y705, T714, and S727) in Ki-JK and SUDHL-1 cells using the phospho-specific antibodies. As a result, treatment with either crizotinib or alectinib markedly reduced the phosphorylation of NPM–ALK, as well as the phosphorylation levels at all three STAT3 sites (Y705, T714, and S727) in ALCL patient-derived cell lines (Fig. 2A, B).

**Fig. 3:**
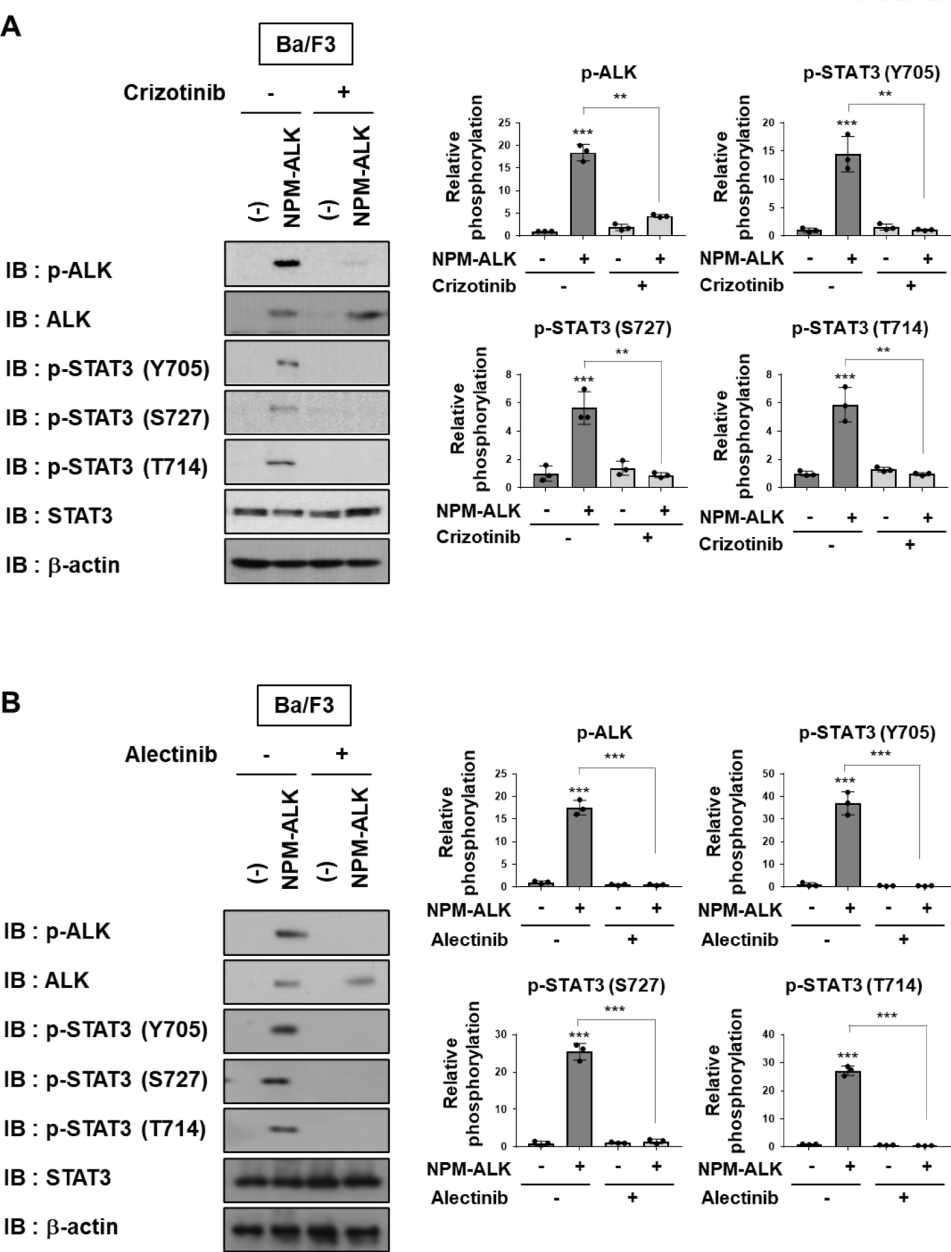
The enforced expression of NPM-ALK induces the phosphorylation of STAT3 at Y705, T714, and S727 in a kinase activity-dependent manner in Ba/F3 cells. Ba/F3 cells were infected with an empty virus (–) or a retrovirus expressing NPM-ALK. Cells were treated with (**A**) 1 µM crizotinib or (**B**) 0.5 µM alectinib for 24 h. Whole-cell lysates were prepared and subjected to immunoblotting. The membranes were cut prior to antibody incubation. ALK and STAT3 phosphorylation levels were quantified (*n* = 3). The significance of differences was set as **p < 0.01, ***p < 0.001. All original blot images, including all replicates, are provided in the Supplementary Information.

To further examine the phosphorylation status of STAT3 induced by NPM-ALK, control Ba/F3 cells infected with an empty vector (−) and Ba/F3 cells expressing NPM-ALK were treated with either crizotinib or alectinib, and STAT3 phosphorylation was then evaluated by immunoblotting. In Ba/F3 cells expressing NPM-ALK, the strong phosphorylation of NPM-ALK itself as well as STAT3 at Y705, T714, and S727 was observed. These phosphorylation events were effectively suppressed by treatment with either crizotinib or alectinib (Fig. 3A, B). Taken together, these results suggest that STAT3 phosphorylation at T714, along with Y705 and S727, is mediated by NPM-ALK in a kinase activity–dependent manner.

### The NPM-ALK–induced phosphorylation of STAT3 at T714 contributes to the subsequent phosphorylation of STAT3 at Y705

To investigate the functional consequence of T714 phosphorylation observed in human ALCL cells, we utilized the murine Ba/F3 cell line expressing NPM-ALK as a model system. We first performed STAT3 knockdown using shRNA in Ba/F3 cells expressing NPM-ALK and evaluated the efficiency and durability of STAT3 suppression. Immunoblotting and RT-PCR analyses confirmed that STAT3 expression was sustainably suppressed at both the protein and mRNA levels for up to 96 h after puromycin removal in the infected Ba/F3 cells (Supplemental Fig. 1).

**Fig. 4:**
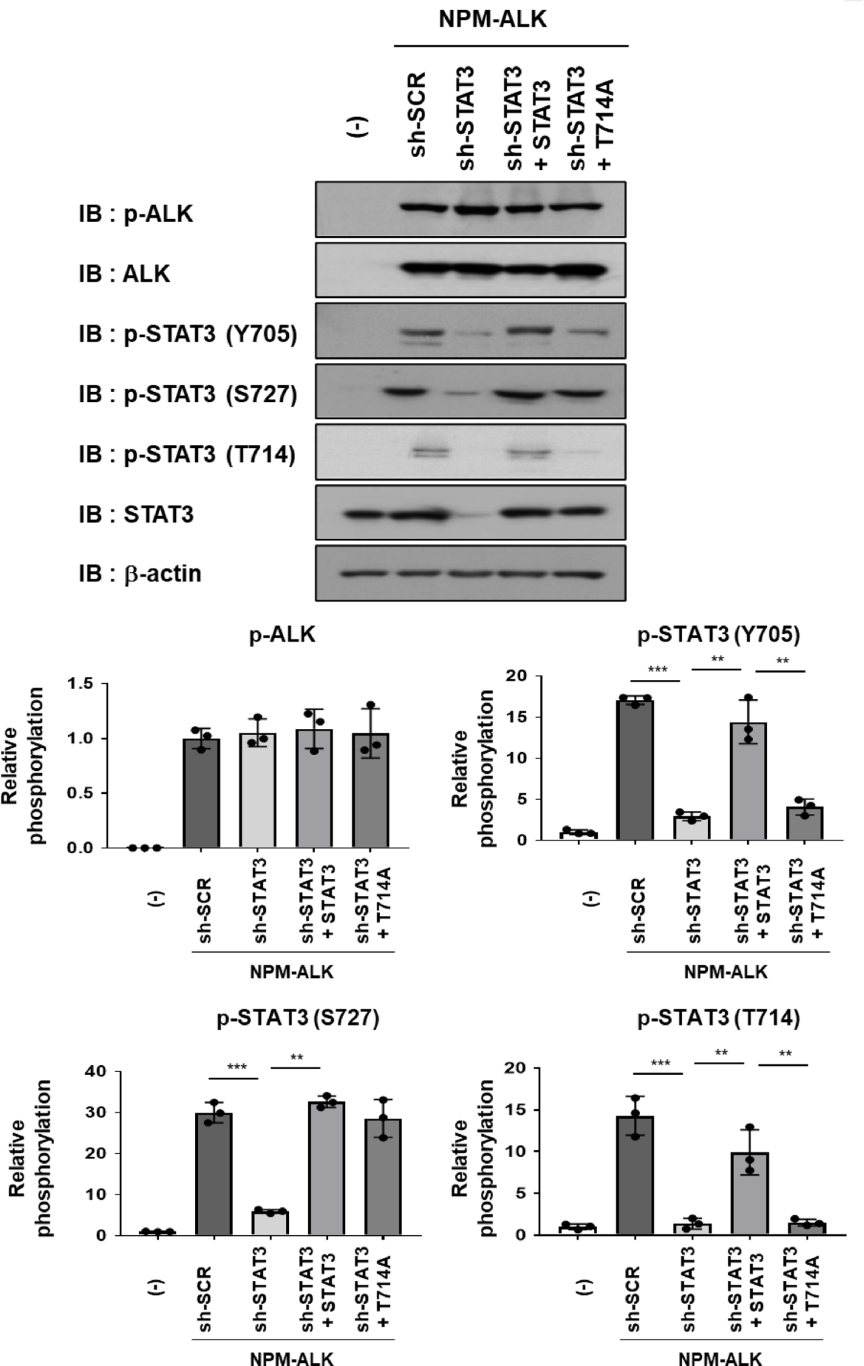
The reconstituted STAT3 T714A mutant is phosphorylated at S727, but not at Y705 in Ba/F3 cells expressing NPM-ALK and sh-STAT3. Ba/F3 cells were infected with an empty virus (–) or a retrovirus expressing NPM-ALK. Ba/F3 cells expressing NPM-ALK were further transduced with either sh-scramble (sh-SCR) or shRNA targeting STAT3 (sh-STAT3) via retroviral infection. Ba/F3 cells expressing NPM-ALK and sh-STAT3 were then infected with retroviruses expressing wild-type STAT3 or the T714A mutant. Transduced cells were incubated in RPMI medium containing 10% FBS for 24 h. Whole-cell lysates were prepared and subjected to immunoblotting. The membranes were cut prior to antibody incubation. ALK and STAT3 phosphorylation levels were quantified (*n* = 3). The significance of differences was set as **p < 0.01, ***p < 0.001. All original blot images, including all replicates, are provided in the Supplementary Information.

Subsequently, to investigate the role of STAT3 phosphorylation at T714, we generated a STAT3 mutant (T714A) in which threonine 714 was substituted with alanine. Ba/F3 cells expressing NPM-ALK were infected with a retrovirus encoding shRNA targeting STAT3 to knockdown endogenous STAT3, followed by reconstitution with either wild-type STAT3 or the T714A mutant. The knockdown of STAT3 and reconstitution with wild-type or T714A mutant STAT3 did not affect the phosphorylation or expression levels of NPM-ALK. In reconstituted cells, wild-type STAT3 was phosphorylated at both Y705 and S727, whereas the T714A mutant was phosphorylated only at S727, with Y705 phosphorylation being markedly suppressed (Fig. 4). These results suggest that phosphorylation at T714 was a prerequisite for the NPM-ALK–dependent phosphorylation of STAT3 at Y705 in this model.

### STAT3 phosphorylation at T714 contributes to its NPM-ALK–induced nuclear translocation

**Fig. 5:**
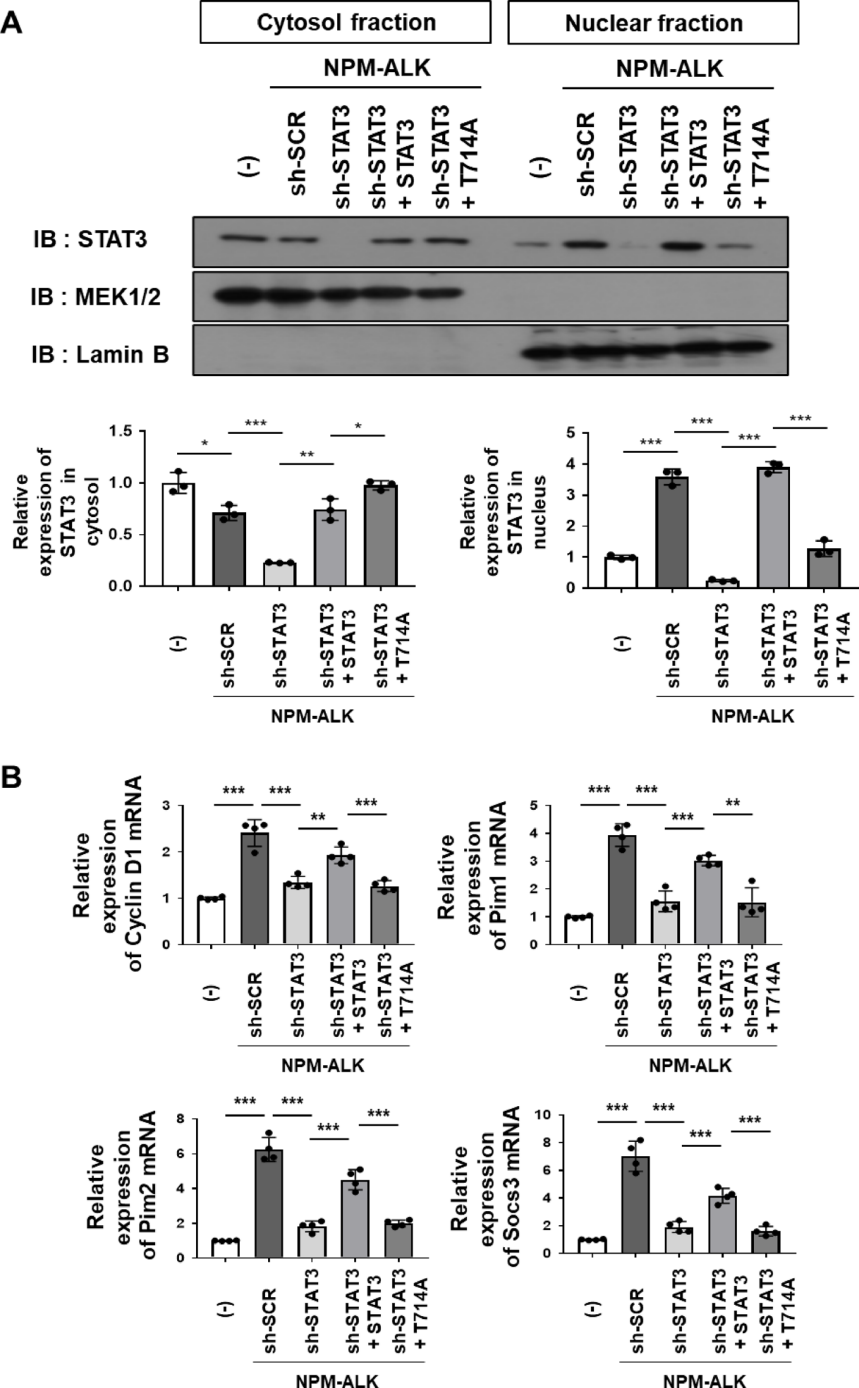
The reconstituted STAT3 T714A mutant fails to translocate into the nucleus and induce the expression of STAT3 target genes in Ba/F3 cells co-expressing NPM-ALK and sh-STAT3. Transduced cells were incubated in RPMI medium containing 10% FBS for 24 h. (**A**) Cytosolic and nuclear fractions were isolated and subjected to immunoblotting. The membranes were cut prior to antibody incubation. The expression levels of STAT3 and its mutant in the cytosolic and nuclear compartments were quantified (*n* = 3). The significance of differences was set as *p < 0.05, **p < 0.01, ***p < 0.001. All original blot images, including all replicates, are provided in the Supplementary Information. (**B**) Total RNA was extracted and RT-PCR was performed. The relative mRNA expression levels of Cyclin D1, Pim1, Pim2, and Socs3 were calculated (*n* = 3). The significance of differences was set as **p < 0.01, ***p < 0.001.

Activated STAT3 is translocated into the nucleus to regulate gene expression^13,14^. Since STAT phosphorylation at T714 appeared to affect that at Y705, we investigated its effects on the nuclear translocation of STAT3 downstream of NPM-ALK signaling. Because Ba/F3 cells are suspension cells, immunofluorescence-based localization was technically challenging. Therefore, to evaluate the nuclear translocation of wild-type and mutant STAT3 proteins, we prepared cytoplasmic and nuclear fractions and performed immunoblot analysis. In control Ba/F3 cells (−), STAT3 was confined to the cytoplasm. In Ba/F3 cells expressing NPM-ALK and sh-scramble (sh-SCR), STAT3 was detected in both the cytoplasm and nucleus. Similarly, reconstituted wild-type STAT3 localized to both compartments in Ba/F3 cells expressing NPM-ALK with sh-STAT3. In contrast, the T714A mutant remained predominantly in the cytoplasm (Fig. 5A). These results indicate that STAT3 phosphorylation at T714 contributes not only to its phosphorylation at Y705, but also to its nuclear translocation driven by NPM-ALK signaling.

To assess the impact of T714 phosphorylation on STAT3 transcriptional activity, we performed a RT-PCR analysis of representative STAT3 target genes, including Cyclin D1, Pim1, Pim2, and Socs3^24,27–30^. *Cyclin D1* encodes a cell cycle regulator that promotes G1/S phase progression^31^, *Pim1* and *Pim2* encode serine/threonine kinases that enhance cell survival and proliferation^32,33^, and *Socs3* encodes a suppressor of cytokine signaling that negatively regulates the JAK/STAT pathway^34,35^.

The mRNA levels of these genes were significantly higher in Ba/F3 cells expressing NPM-ALK than in control Ba/F3 cells (−). Moreover, their expression was markedly reduced upon STAT3 knockdown, but was efficiently restored by reconstitution with wild-type STAT3. In contrast, reconstitution with the T714A mutant of STAT3 failed to restore their expression (Fig. 5B). These results suggest that the phosphorylation of STAT3 at T714 contributes to its transcriptional activation mediated by NPM-ALK signaling in the Ba/F3 system.

### STAT3 phosphorylation at T714 contributes to NPM-ALK–induced cell proliferation

**Fig. 6:**
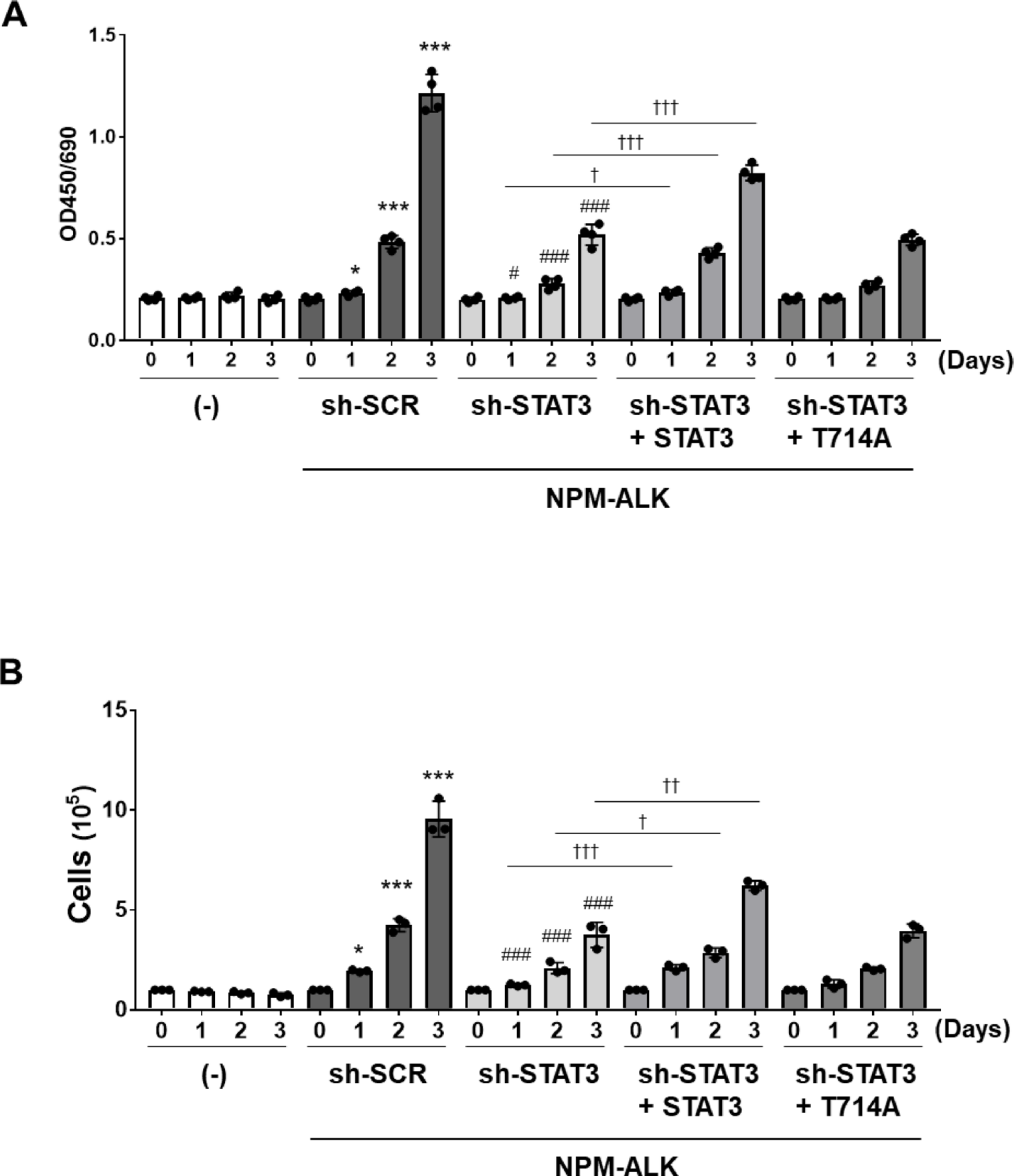
The reconstituted STAT3 T714A mutant fails to restore proliferation in Ba/F3 cells co-expressing NPM-ALK and sh-STAT3. (**A**,**B**) Transduced Ba/F3 cells were incubated with RPMI medium containing 10% FBS for 3 days. (**A**) Cell proliferation was examined by a WST assay. Data are shown as the mean ± SD of four independent experiments. The significance of differences was set as follows: *p < 0.05, ***p < 0.001 vs. control cells (–); #*p* < 0.05, ###*p* < 0.001 vs. Ba/F3 cells expressing NPM-ALK and sh-scramble (sh-SCR); †*p* < 0.05, †††*p* < 0.001 vs. Ba/F3 cells expressing sh-STAT3 reconstituted with STAT3. (**B**) Viable cells were counted by trypan blue staining. Data are shown as the mean ± SD of three independent experiments. The significance of differences was set as follows: *p < 0.05, ***p < 0.001 vs. control cells (–); ###*p* < 0.001 vs. Ba/F3 cells expressing NPM-ALK and sh-scramble (sh-SCR); †*p* < 0.05, ††*p* < 0.01, †††*p* < 0.001 vs. Ba/F3 cells expressing sh-STAT3 reconstituted with STAT3.

The enforced expression of NPM-ALK confers a cytokine-independent proliferative capacity to Ba/F3 cells^8^. To investigate the roles of STAT3 and its phosphorylation at T714 in NPM-ALK–induced cell proliferation, we performed WST assays. The knockdown of STAT3 significantly suppressed the proliferation of Ba/F3 cells expressing NPM-ALK. Reconstitution with wild-type STAT3 restored the proliferative capacity of cells, whereas reconstitution with the T714A mutant did not (Fig. 6A).

We also measured viable cells by trypan blue staining. The increase in the number of Ba/F3 cells expressing NPM-ALK was significantly reduced by the knockdown of STAT3 and restored by the reconstitution of STAT3, but not T714A (Fig. 6B).

**Fig. 7:**
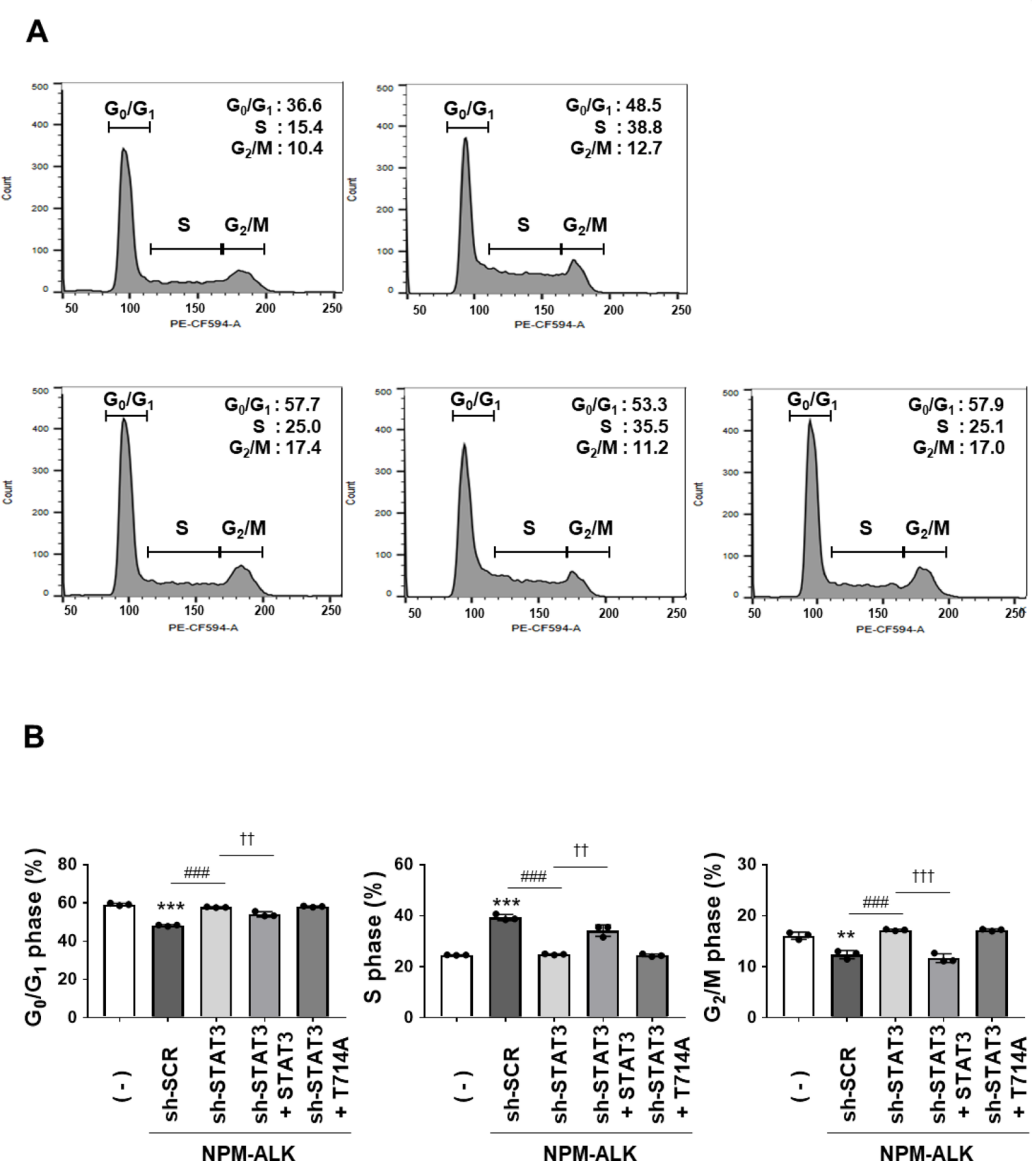
The reconstituted STAT3 T714A mutant fails to restore normal cell cycle progression in Ba/F3 cells co-expressing NPM-ALK and sh-STAT3. Transduced cells were incubated with RPMI medium containing 10% FBS for 24 h. After cells were fixed with 70% EtOH, cell cycle distributions were assessed by PI staining. (**A**) Representative histograms of cell cycle distribution obtained by PI staining. (**B**) Data are shown as the mean ± SD of three independent experiments. The significance of differences was set as follows: **p < 0.01, ***p < 0.001 vs. control cells (–); ###*p* < 0.001, vs. Ba/F3 cells expressing NPM-ALK and sh-scramble (sh-SCR); ††*p* < 0.01, †††*p* < 0.001 vs. Ba/F3 cells expressing sh-STAT3 reconstituted with STAT3.

We next investigated the cell cycle distribution in these cells. In comparison with control Ba/F3 cells (−), Ba/F3 cells expressing NPM-ALK transduced with sh-SCR showed a decreased percentage of cells in the G_0_/G_1_ phase and a marked increase of those in the S phase. Upon STAT3 knockdown, Ba/F3 cells expressing NPM-ALK showed an increased G_0_/G_1_ population and decreased S phase population. Reconstitution with wild-type STAT3 significantly reversed these changes, whereas reconstitution with the T714A mutant did not affect the cell cycle distribution in STAT3-knockdown Ba/F3 cells expressing NPM-ALK (Fig. 7A,B). These results suggest that the phosphorylation of STAT3 at T714 contributes to NPM-ALK-induced cell proliferation. 

### The phosphorylation of STAT3 at T714 contributes to NPM-ALK-induced tumorigenesis

**Fig. 8:**
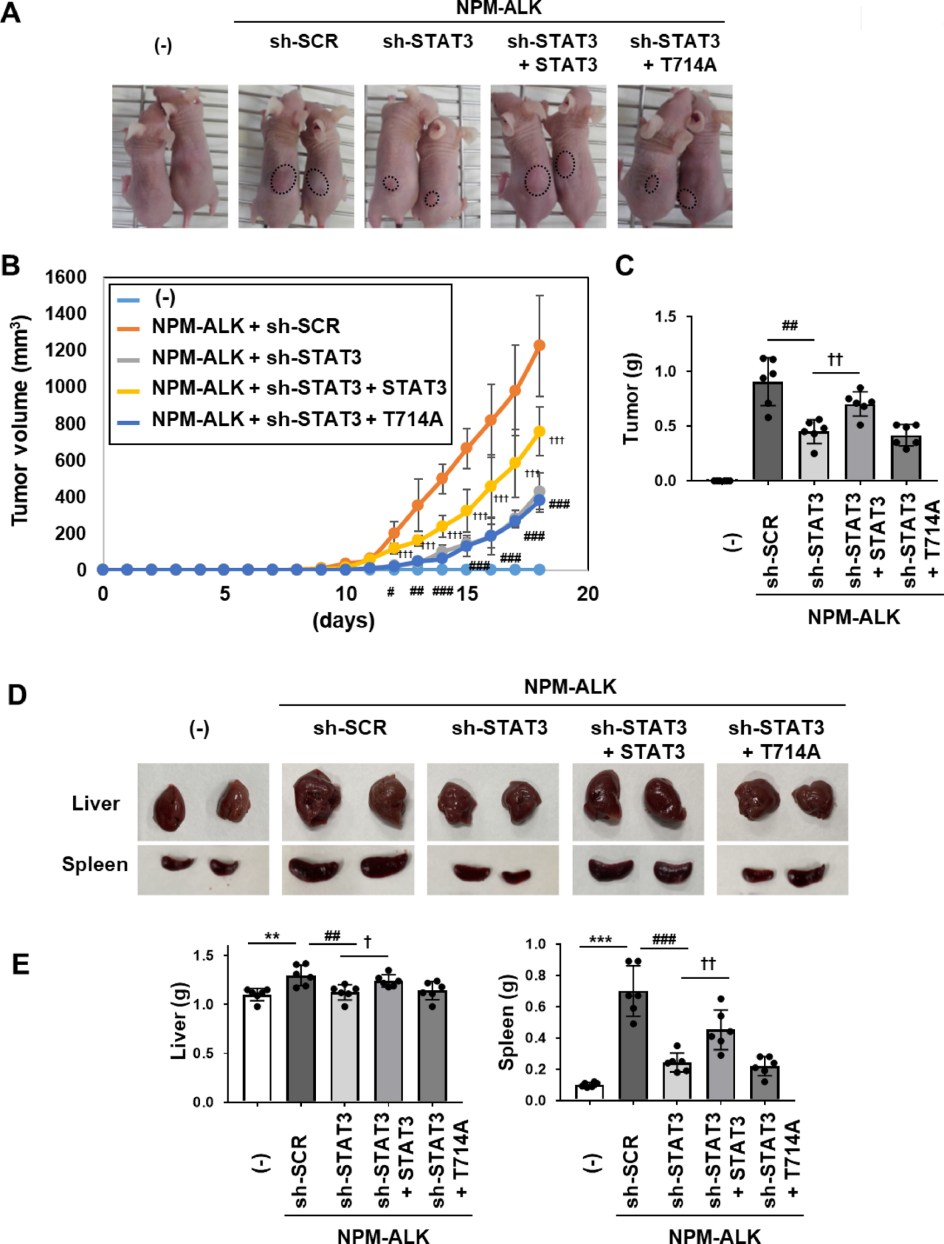
The reconstituted STAT3 T714A mutant does not induce tumor formation or hypertrophy of the liver and spleen in mice inoculated with Ba/F3 cells expressing NPM-ALK and sh-STAT3. Transduced Ba/F3 cells (1 × 10^7^ cells) were subcutaneously injected into nude mice (*n* = 6). (**A**) Eighteen days after the inoculation, mice were photographed and dissected. (**B**) From 8 days after the inoculation, tumor volumes were measured for 10 days. (**C**) The weights of tumors were measured. The significance of differences was set as follows: ##*p* < 0.01, vs. Ba/F3 cells expressing NPM-ALK and sh-scramble (sh-SCR); ††*p* < 0.01 vs. Ba/F3 cells expressing sh-STAT3. (**D**) Morphological changes in spleens and livers were photographed. (**E**) The weights of spleens and livers were measured. The significance of differences was set as follows: **p < 0.01, ***p < 0.001 vs. control cells (–); ##*p* < 0.01, ###*p* < 0.001, vs. Ba/F3 cells expressing NPM-ALK and sh-scramble (sh-SCR); †*p* < 0.05, ††*p* < 0.01 vs. Ba/F3 cells expressing sh-STAT3 reconstituted with STAT3.

To investigate the effects of STAT3 phosphorylation at T714 on NPM-ALK–induced tumorigenesis, control Ba/F3 cells (−), Ba/F3 cells expressing NPM-ALK with sh-SCR or sh-STAT3, and cells reconstituted with wild-type STAT3 or the T714A mutant were subcutaneously implanted into nude mice. While the inoculation with control Ba/F3 cells (−) did not result in tumor formation, Ba/F3 cells expressing NPM-ALK induced significant tumorigenesis in nude mice. Importantly, tumor formation was markedly suppressed by STAT3 knockdown, indicating that STAT3 was required for NPM-ALK–mediated tumorigenic activity (Fig. 8A). Tumor length and width were measured daily starting on day 8 post-inoculation, and tumor volume was calculated accordingly (Fig. 8B, Supplemental Table 1)^26^. In comparison with mice inoculated with Ba/F3 cells expressing NPM-ALK and sh-SCR, increases in both tumor volume and tumor weight were significantly attenuated in mice inoculated with Ba/F3 cells expressing NPM-ALK and sh-STAT3. Furthermore, in mice inoculated with Ba/F3 cells expressing NPM-ALK and sh-STAT3, reconstitution with wild-type STAT3—but not with the T714A mutant—significantly restored tumor growth, as evidenced by increases in both tumor volume and weight (Fig. 8B, C).

The inoculation of Ba/F3 cells expressing NPM-ALK and sh-SCR induced hypertrophy of the liver and spleen (Fig. 8D). Measurements of the weights of the liver and spleen in each mouse revealed that the inoculation of Ba/F3 cells expressing NPM-ALK and sh-SCR significantly increased both weights, while the inoculation of Ba/F3 cells expressing NPM-ALK and sh-STAT3 significantly decreased both weights. In addition, the reconstitution with STAT3, but not T714A, increased the weights of the livers and spleens in nude mice inoculated with Ba/F3 cells expressing NPM-ALK and sh-STAT3 (Fig. 8E, Supplementary Table 2).

Collectively, these results suggest that the phosphorylation of STAT3 at T714 plays a critical role in NPM-ALK-induced tumorigenic potential in this experimental model.

## Discussion

We herein investigated the biochemical role of STAT3 phosphorylation in the NPM-ALK-mediated signaling pathway. We previously demonstrated that STAT3 phosphorylation at Y705 in the downstream signaling pathway of NPM-ALK was essential for transcriptional activation^23^. In the present study, we newly identify phosphorylation at T714 as a previously uncharacterized modification required for efficient Y705 phosphorylation and maximal STAT3 activation downstream of NPM-ALK.

**Fig. 9:**
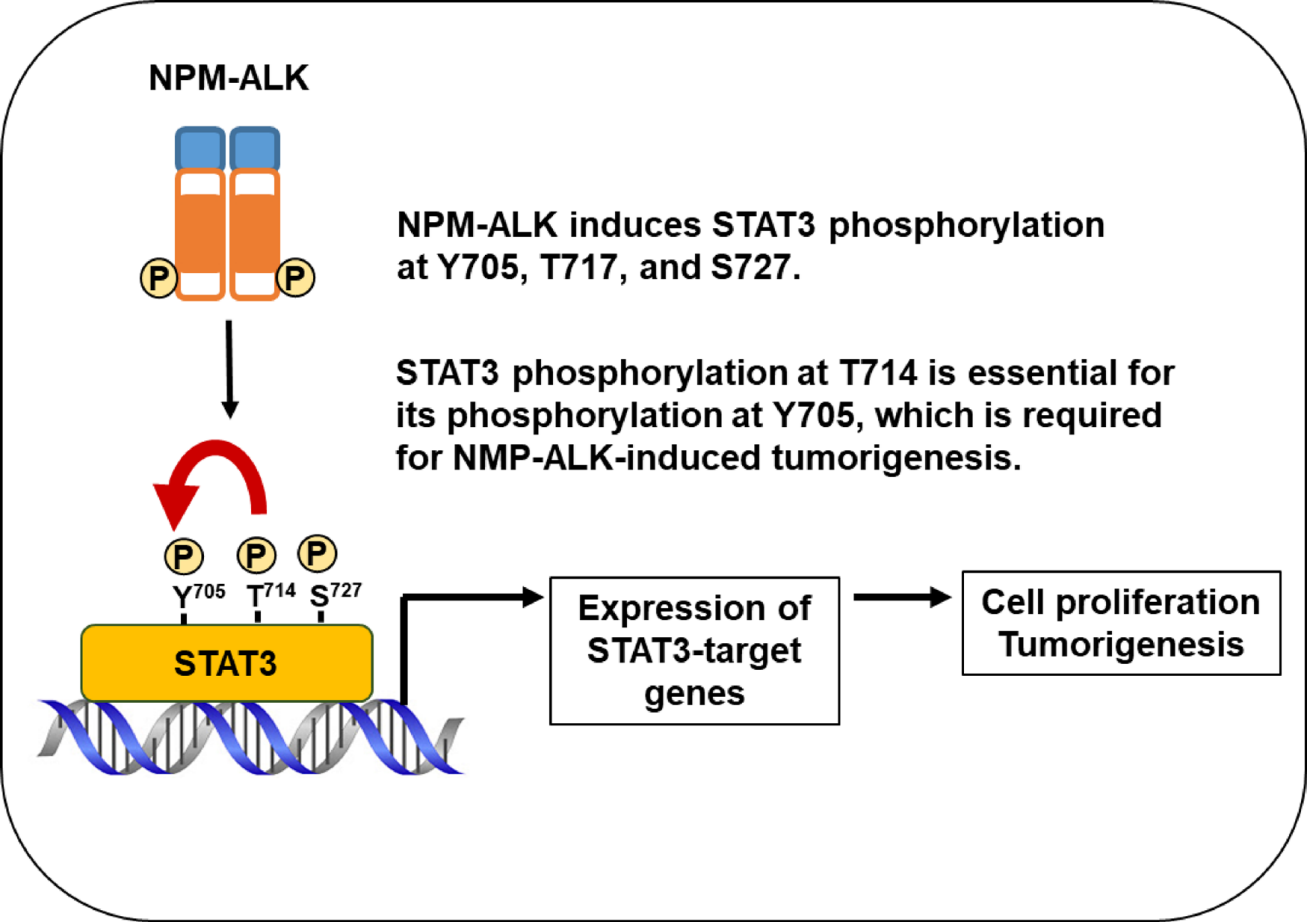
The phosphorylation of STAT3 at T714 contributes to the NPM-ALK-induced activation of STAT3, cell proliferation, and tumorigenesis. NPM-ALK induces the phosphorylation of STAT3 at Y705, T714, and S727 in a kinase activity-dependent manner. Phosphorylation at T714 facilitates subsequent Y705 phosphorylation, which is important for STAT3 activation, and plays a critical role in NPM-ALK–mediated cell proliferation and tumorigenesis.

Our results indicate that NPM-ALK promotes STAT3 phosphorylation at Y705, S727, and T714 in a kinase-dependent manner in ALCL patient-derived cells. Analysis using the Ba/F3 model revealed that T714 phosphorylation contributes to subsequent Y705 modification and nuclear translocation. The observation that the T714A mutant retained S727 phosphorylation but failed to restore Y705 phosphorylation or transcriptional activity supports a hierarchical phosphorylation model, in which T714 functions as a molecular trigger for STAT3 activation. This regulatory framework underscores the importance of coordinated phosphorylation events in fine-tuning STAT3 function and highlights T714 as a critical regulatory node within NPM-ALK–driven signaling (Fig. 9).

This hierarchical phosphorylation mechanism appears to be dependent on the three-dimensional structure of STAT3 and cooperative interactions among its phosphorylation sites. Similar examples of coordinated phosphorylation within a single molecule have been reported for extracellular signal-regulated kinase (ERK) and p53. In ERK, dual phosphorylation at T183 and Y185 within the activation loop was essential for stabilizing the active conformation and enabling substrate recognition^36^. In p53, phosphorylation at S15, T18, and S20 disrupted MDM2 binding and promoted protein stabilization, while phosphorylation at T55 functioned as a switch that modulated DNA-binding cooperativity^37,38^. These findings suggest that phosphorylation functions not only as an activation signal, but also as a structural and functional modulator. In this context, phosphorylation at T714 may induce conformational changes in STAT3 that promote Y705 phosphorylation and nuclear translocation.

Supporting this idea, Waitkus et al. demonstrated that co-stimulation of human umbilical vein endothelial cells with EGFR and PAR-1 induced STAT3 phosphorylation at T714 and S727, without detectable Y705 phosphorylation. Further mechanistic studies showed that recombinant GSK3β directly phosphorylated STAT3 at T714 and S727 in vitro. Moreover, siRNA-mediated knockdown of GSK3α and GSK3β significantly reduced EGFR plus PAR-1–induced T714 and S727 phosphorylation, indicating that both isoforms contribute to STAT3 regulation^14^.

GSK3α and GSK3β are highly homologous serine/threonine kinases (~ 98% identity within their catalytic domains) that regulate diverse cellular processes^39,40^. Despite this similarity, they exhibit distinct regulatory roles in cancer. GSK3β is frequently inhibited via the PI3K/AKT pathway in solid tumors, including ALCL, where NPM-ALK activation induces inactivating S9 phosphorylation of GSK3β^41,42^. In contrast, GSK3α remains active in certain cancers and may promote tumorigenesis. For example, in NSCLC, it enhances HIF1/VEGFA signaling under hypoxia^43^. Interestingly, GSK3α inhibition increases GSK3β levels and activity, whereas GSK3α overexpression reduces both, suggesting an isoform-specific regulatory interplay that balances stem cell pluripotency and neural differentiation^44^. Together, these findings suggest that GSK3α may mediate STAT3 T714 phosphorylation even in ALCL, where GSK3β is suppressed. Thus, a key future direction is to determine whether T714 phosphorylation is maintained in a GSK3α-dependent manner in ALCL and other malignancies.

On the other hand, the functional impairment observed in the T714A mutant may not solely result from the loss of phosphorylation but could also involve structural disruption within the linker domain. This region, located between the DNA-binding and SH2 domains, has been reported to stabilize the pY705–SH2 interaction and facilitate proper STAT3 dimerization and nuclear translocation^45,46^. Previous studies have shown that mutations in the linker domain can severely impair STAT3 transcriptional activity, suggesting that it is not merely a structural connector but a functionally active element^47^. Given its structural sensitivity, it is plausible that the T714A mutation disrupts local conformation and interferes with pY705–SH2 engagement, thereby contributing to the observed functional defects. To further clarify the role of T714, future studies using phosphomimetic STAT3 mutants (T714E and T714D) will be essential to determine whether the phenotype of the T714A mutant arises from impaired phosphorylation or structural destabilization.

Although the expression level of reconstituted wild-type STAT3 in STAT3-knockdown Ba/F3 cells expressing NPM-ALK was comparable to that of endogenous STAT3 (Fig. 3), its ability to restore cell proliferation and STAT3 target gene expression was **o**nly partial (Figs. 4 and 5). This modest rescue suggests that exogenously expressed STAT3 may not fully recapitulate the functional properties of the endogenous protein. Possible explanations include differences in post-translational modifications, subcellular localization, or interactions with specific cofactors that may not be fully restored in the rescue setting. These factors should be considered when interpreting the limited functional recovery observed in this context.

However, the physiological significance of T714 phosphorylation within human ALCL cells was not directly addressed in this study. Therefore, the conclusions of this study are focused on defining the biochemical hierarchy of STAT3 phosphorylation specifically downstream of the NPM-ALK oncogene. In future studies, the implementation of inducible shRNA or CRISPR-based systems will be essential to minimize the cytotoxic impact of prolonged STAT3 depletion in human ALCL cell lines. Such approaches will enable functional rescue experiments, allowing for a detailed investigation of the role of T714 within the human ALCL context. Furthermore, to determine the clinical relevance of this modification, analyses using primary ALCL biopsy specimens and genome-wide assessments—such as evaluating chromatin binding and transcriptional output—will be required. Given that ALK rearrangements, such as EML4–ALK, are also present in other malignancies including non–small cell lung cancer (NSCLC)^48^, it will be important to investigate whether T714 phosphorylation represents a conserved regulatory mechanism across diverse ALK-driven tumor types. These subsequent investigations will further clarify the physiological importance of T714 in the pathogenesis of ALK-positive ALCL as a disease entity.

The present study also provides important insights from a therapeutic perspective. Several STAT3 inhibitors, such as Stattic and S3I-201, have been developed to suppress STAT3 activity primarily by inhibiting Y705 phosphorylation or disrupting dimerization via the SH2 domain^49,50^. However, Stattic has been shown to react with N-acetylcysteine (NAC), forming adducts, and its inhibitory activity is suppressed by NAC independently of its antioxidant properties^51^. This finding suggests that Stattic exerts its effects through non-specific interactions with reactive cysteine residues, indicating limitations in its specificity as a STAT3 inhibitor. Therefore, the development of inhibitors targeting T714 phosphorylation, which appears to precede Y705 phosphorylation in STAT3, represents a promising therapeutic strategy to regulate STAT3 activation at an upstream level. Screening for selective inhibitors targeting T714 phosphorylation and identifying the kinase responsible for this modification represent important future directions. These efforts are expected to contribute to the establishment of more precise molecularly targeted therapies directed against STAT3 in the future.

## Supplementary Information

Below is the link to the electronic supplementary material.

Supplementary Material 1

Supplementary Material 2

Supplementary Material 3

Supplementary Material 4

Supplementary Material 5

## Data Availability

Data are available in the article and, when necessary, upon reasonable request fromtago-mg@keio.jp.
