# Supplementary figures and images for: A critical role for STAT3 Thr714 phosphorylation in NPM-ALK-driven tumorigenesis
Source: Sci Rep. 2026 Mar 25;16:15005. doi: 10.1038/s41598-026-44867-w (PMC13172448; doi:10.1038/s41598-026-44867-w)

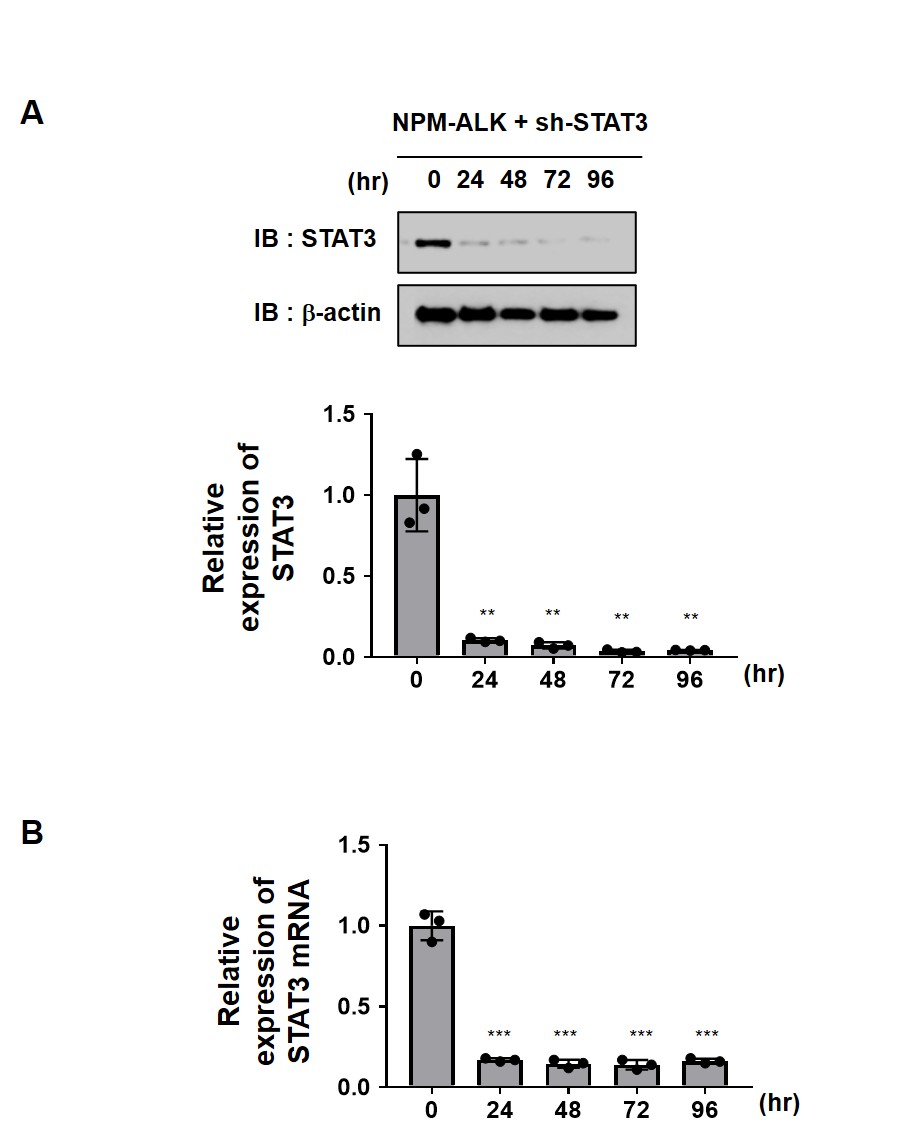

Supplement: Supplementary file 1 — Supplementary Material 1 [file 41598_2026_44867_MOESM1_ESM.jpg]

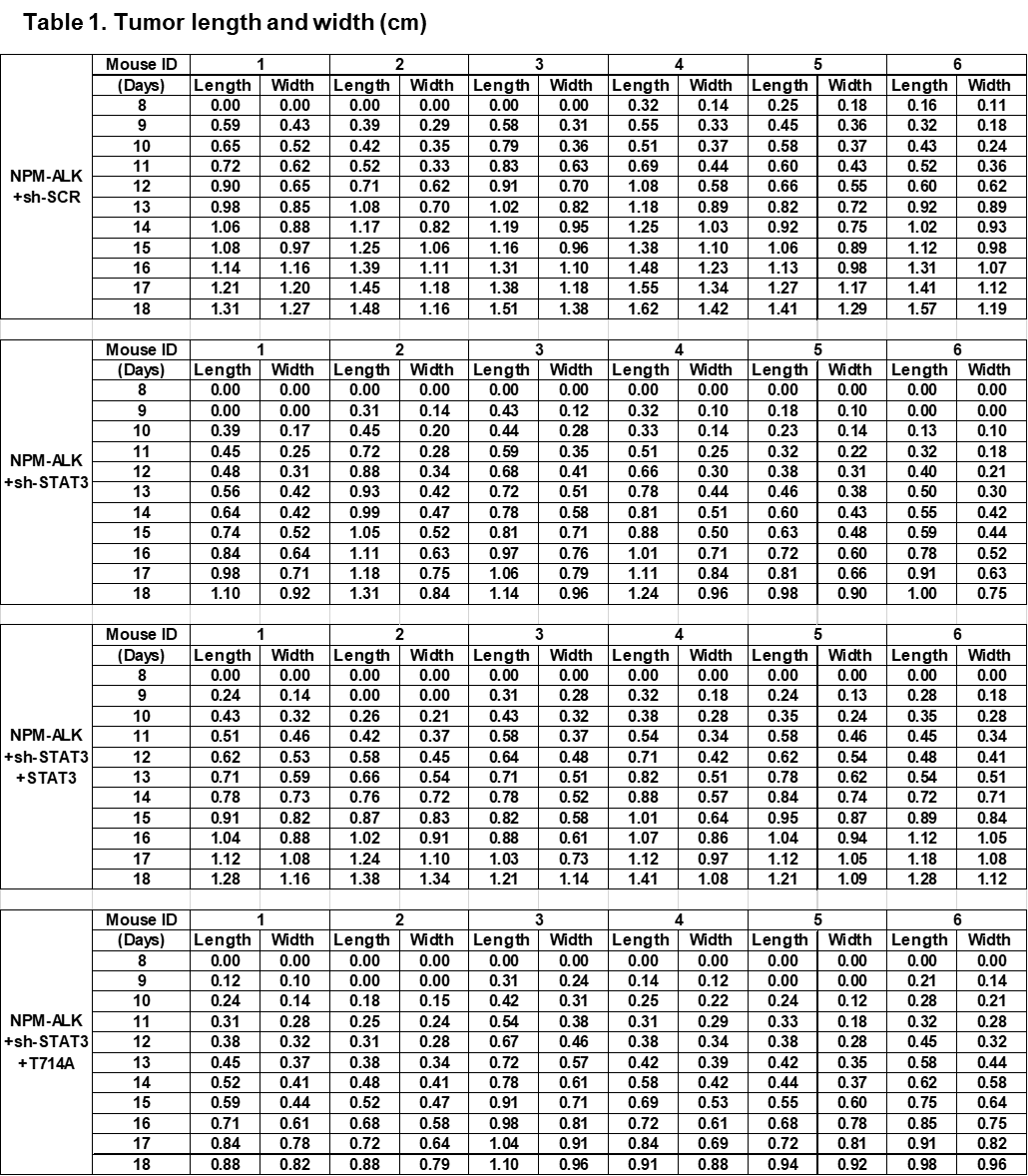


**Supplementary Table 1. Tumor length and width (cm)**

Supplement: Supplementary file 2 — Supplementary Material 2 [file 41598_2026_44867_MOESM2_ESM.docx]
