# Supplementary material for: A critical role for STAT3 Thr714 phosphorylation in NPM-ALK-driven tumorigenesis
Source: Sci Rep. 2026 Mar 25;16:15005. doi: 10.1038/s41598-026-44867-w (PMC13172448; doi:10.1038/s41598-026-44867-w)
Supplement: Supplementary file 3 — Supplementary Material 3 [file 41598_2026_44867_MOESM3_ESM.docx]

**Supplementary Table 2. Tumor, liver, and spleen weights of each mouse.**
