# Supplementary material for: A critical role for STAT3 Thr714 phosphorylation in NPM-ALK-driven tumorigenesis
Source: Sci Rep. 2026 Mar 25;16:15005. doi: 10.1038/s41598-026-44867-w (PMC13172448; doi:10.1038/s41598-026-44867-w)

Original blot images used in Figure 1B

Fig. 1B

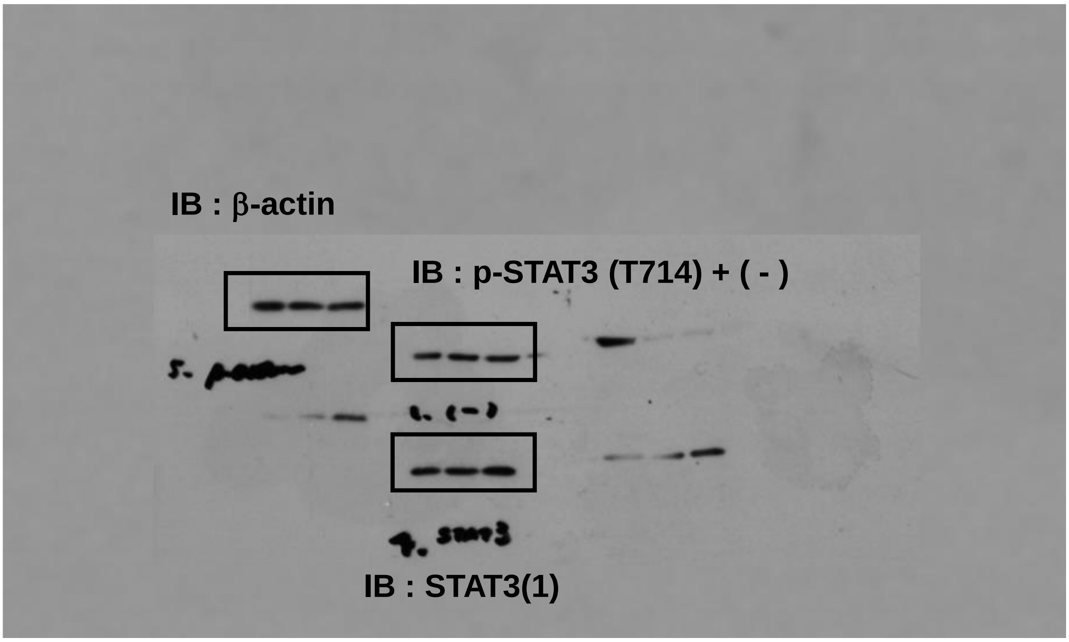

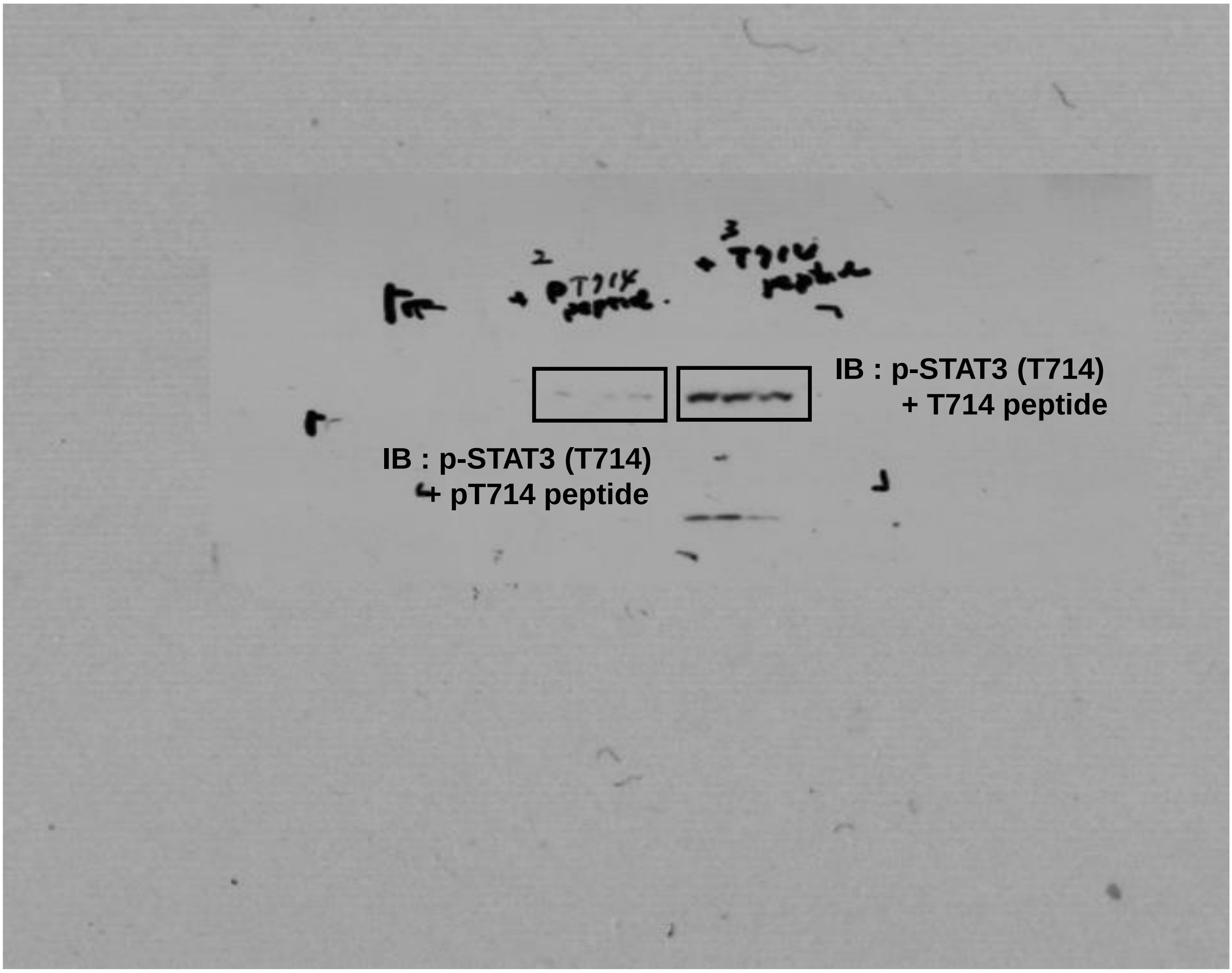

Original blot images, including all replicates used Figure 1C

Fig. 1C

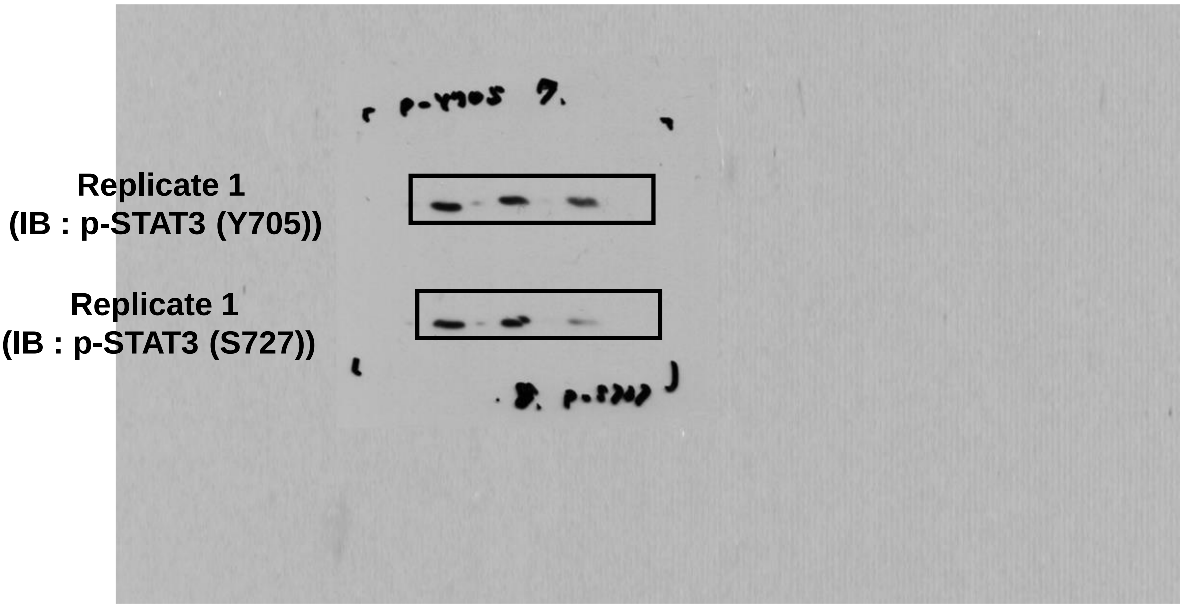

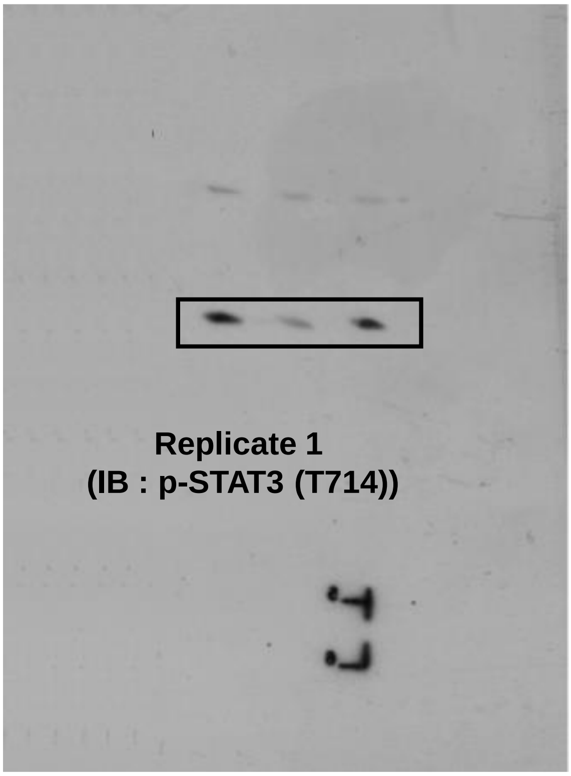

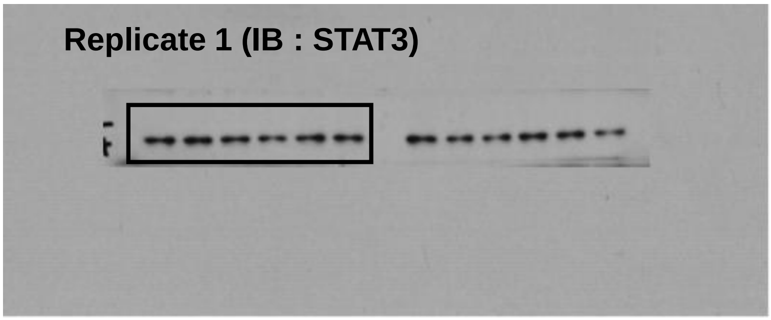

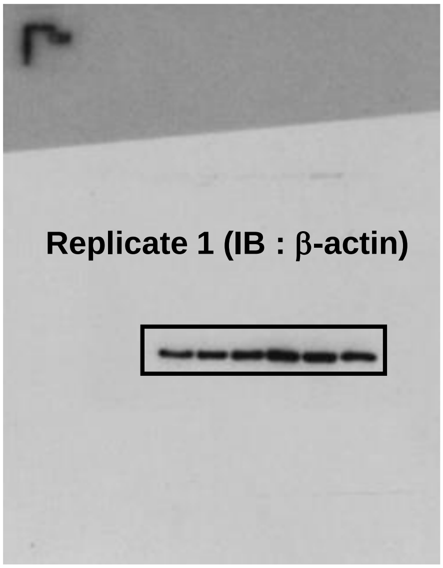

Original blot images, including all replicates used Figure 1C

Fig. 1C

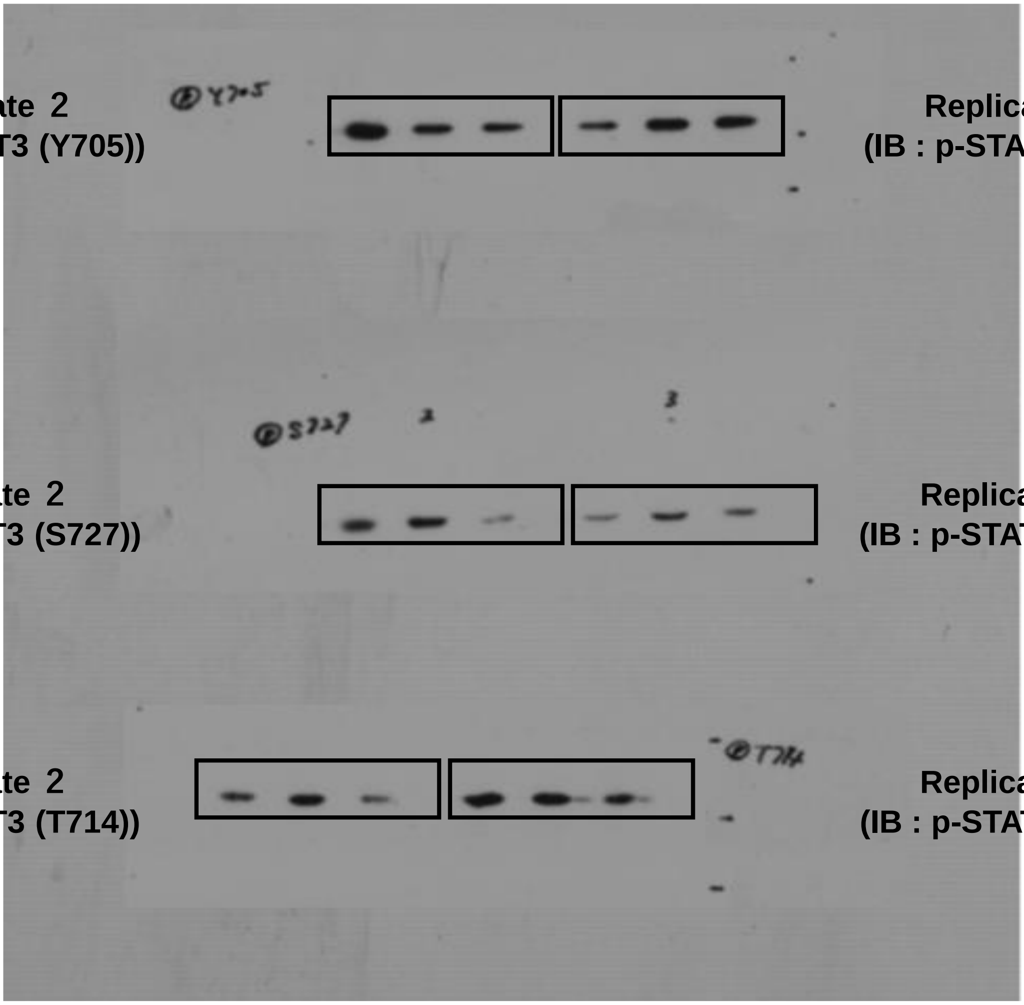

Original blot images, including all replicates used Figure 1C

Fig. 1C

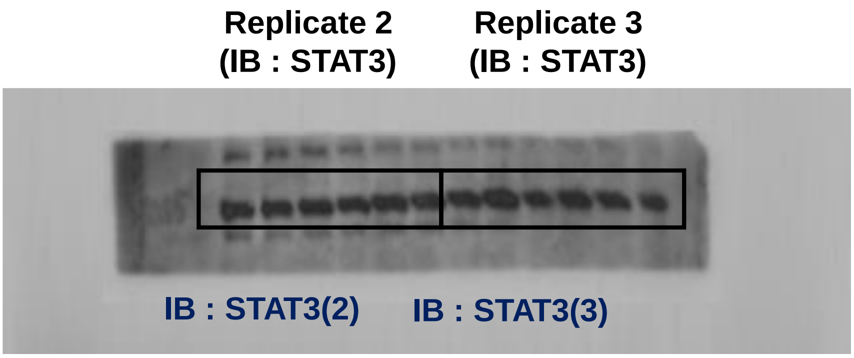

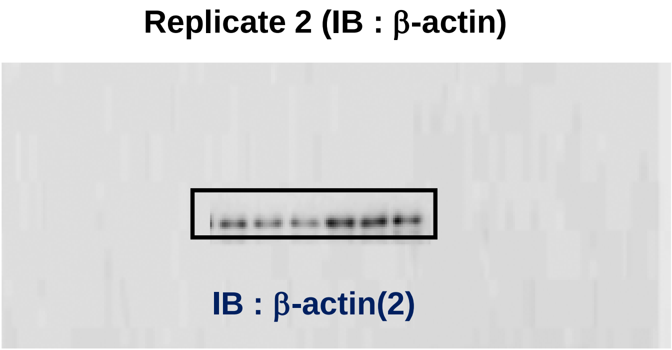

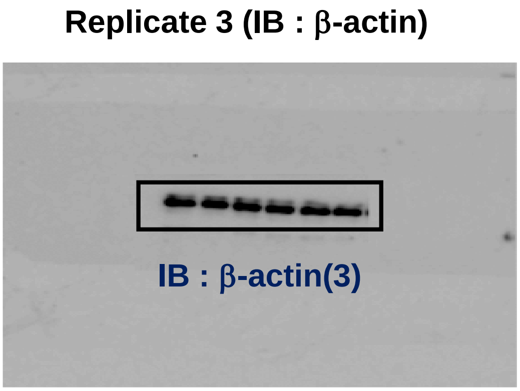

Original blot images, including all replicates used Figure 2A

Fig. 2A

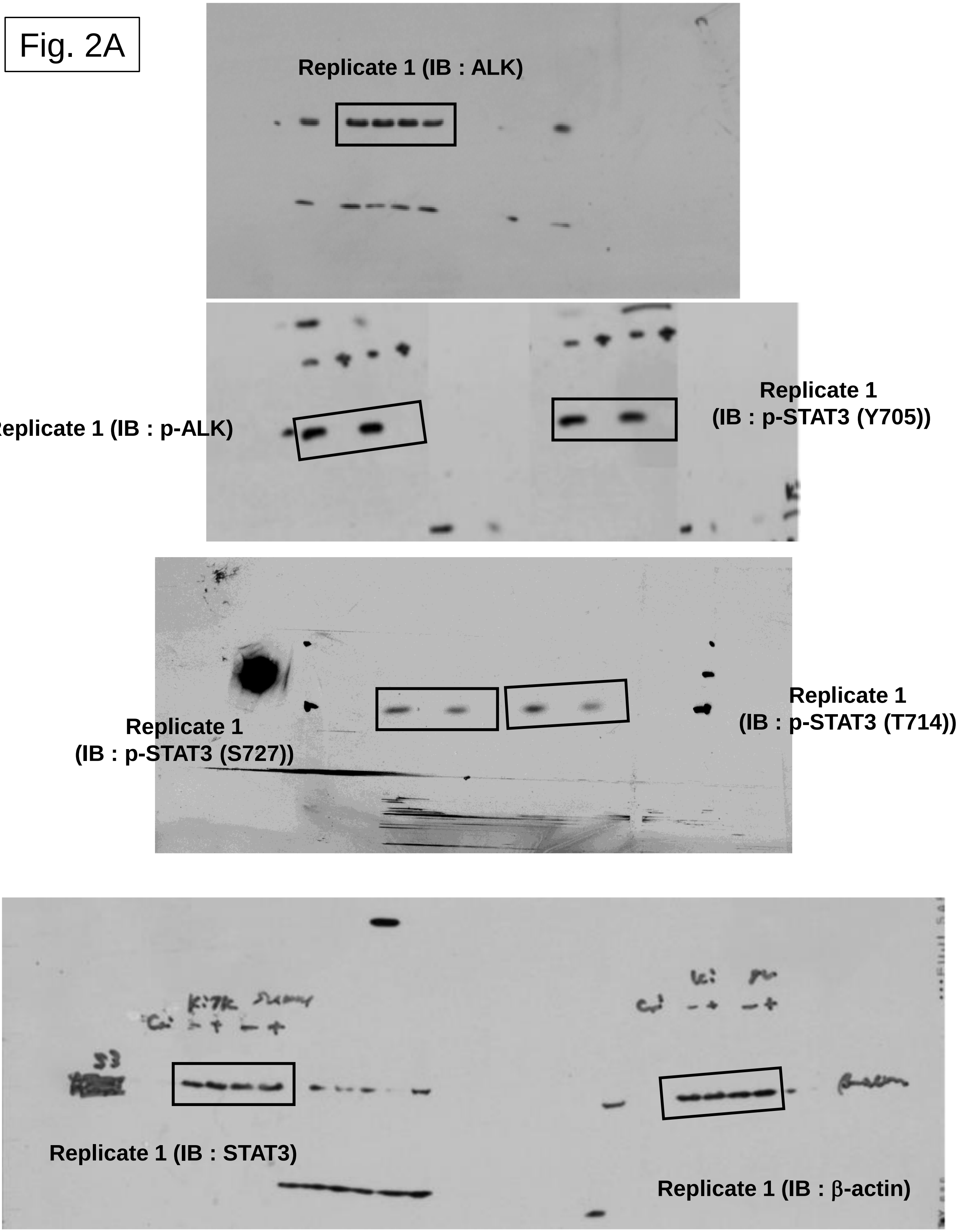

Original blot images, including all replicates used Figure 2A

Fig. 2A

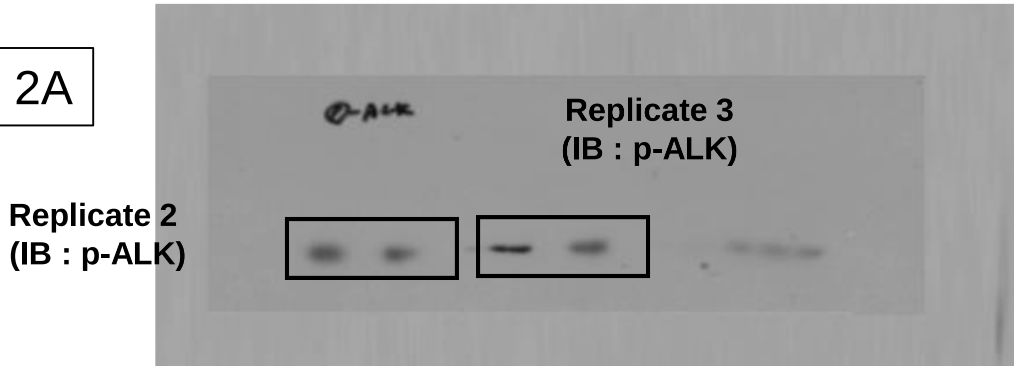

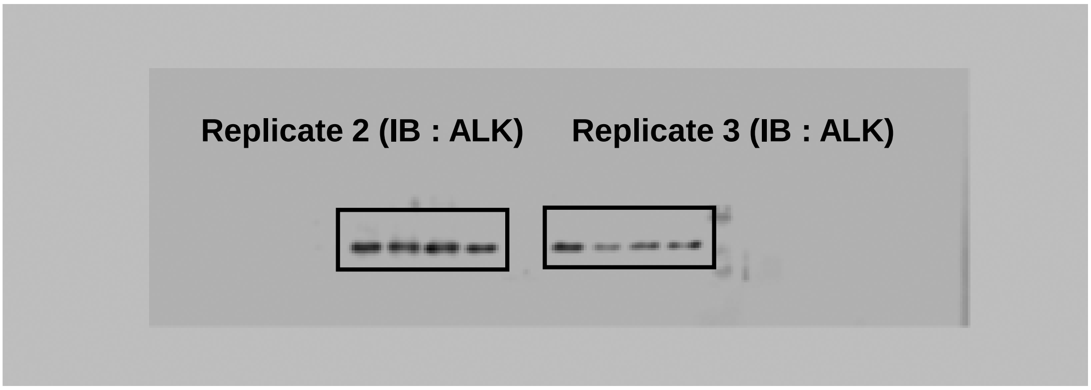

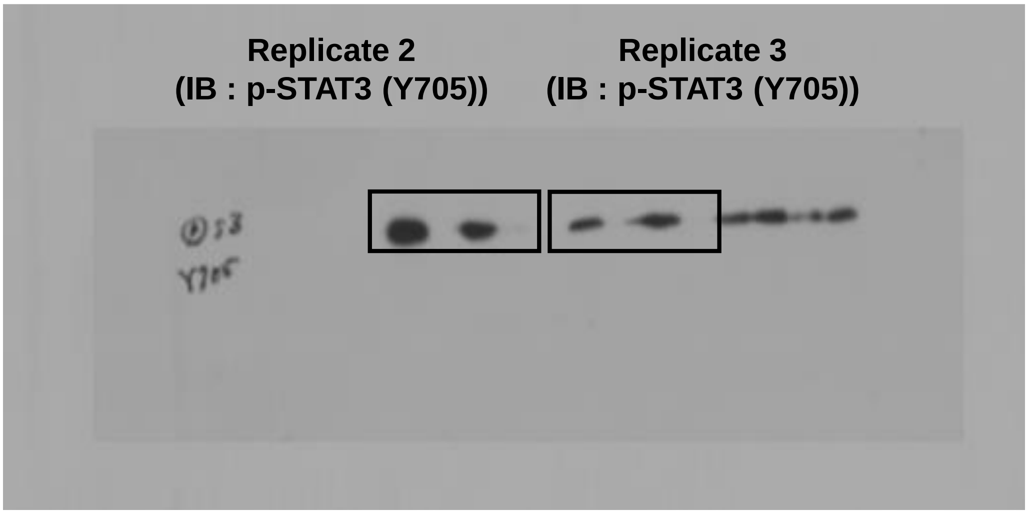

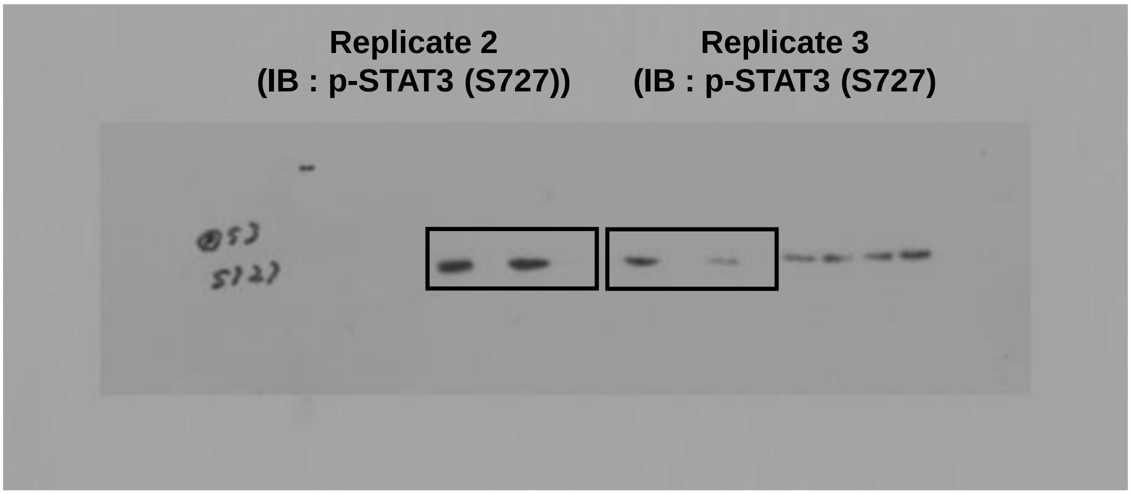

Original blot images, including all replicates used Figure 2A

Fig. 2A

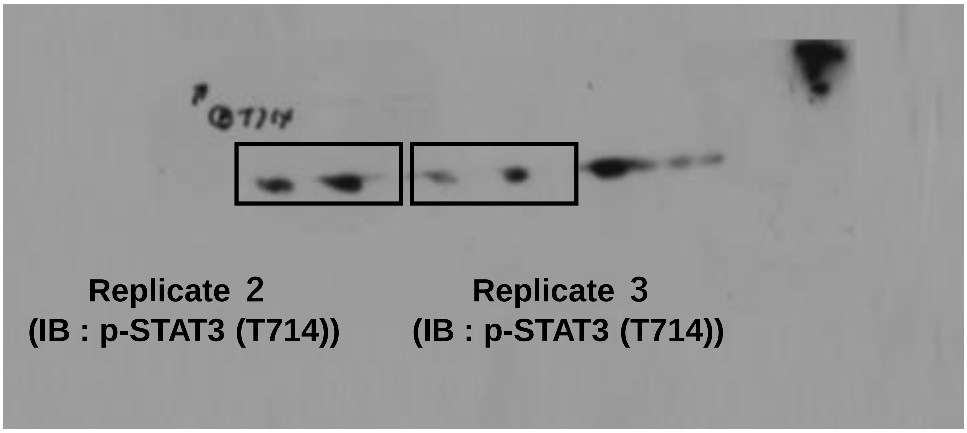

Replicate 2
(IB : STAT3)

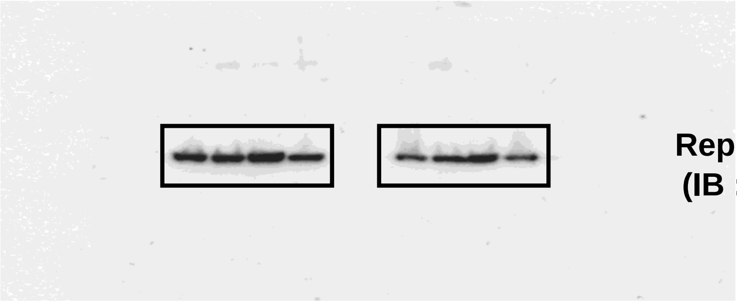

Replicate 3
(IB : STAT3)

Replicate 2
(IB : β-actin)

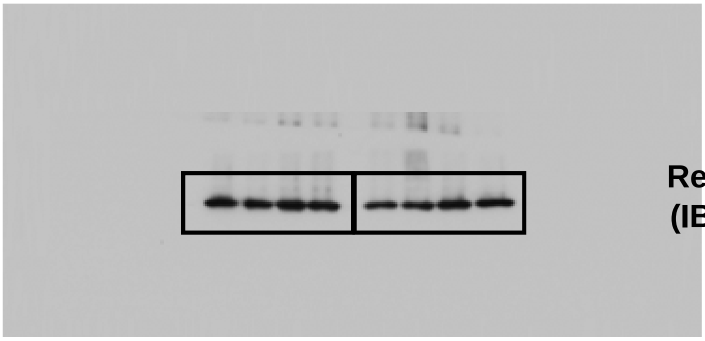

Replicate 3
(IB : β-actin)

Original blot images, including all replicates used Figure 2B

Fig. 2B

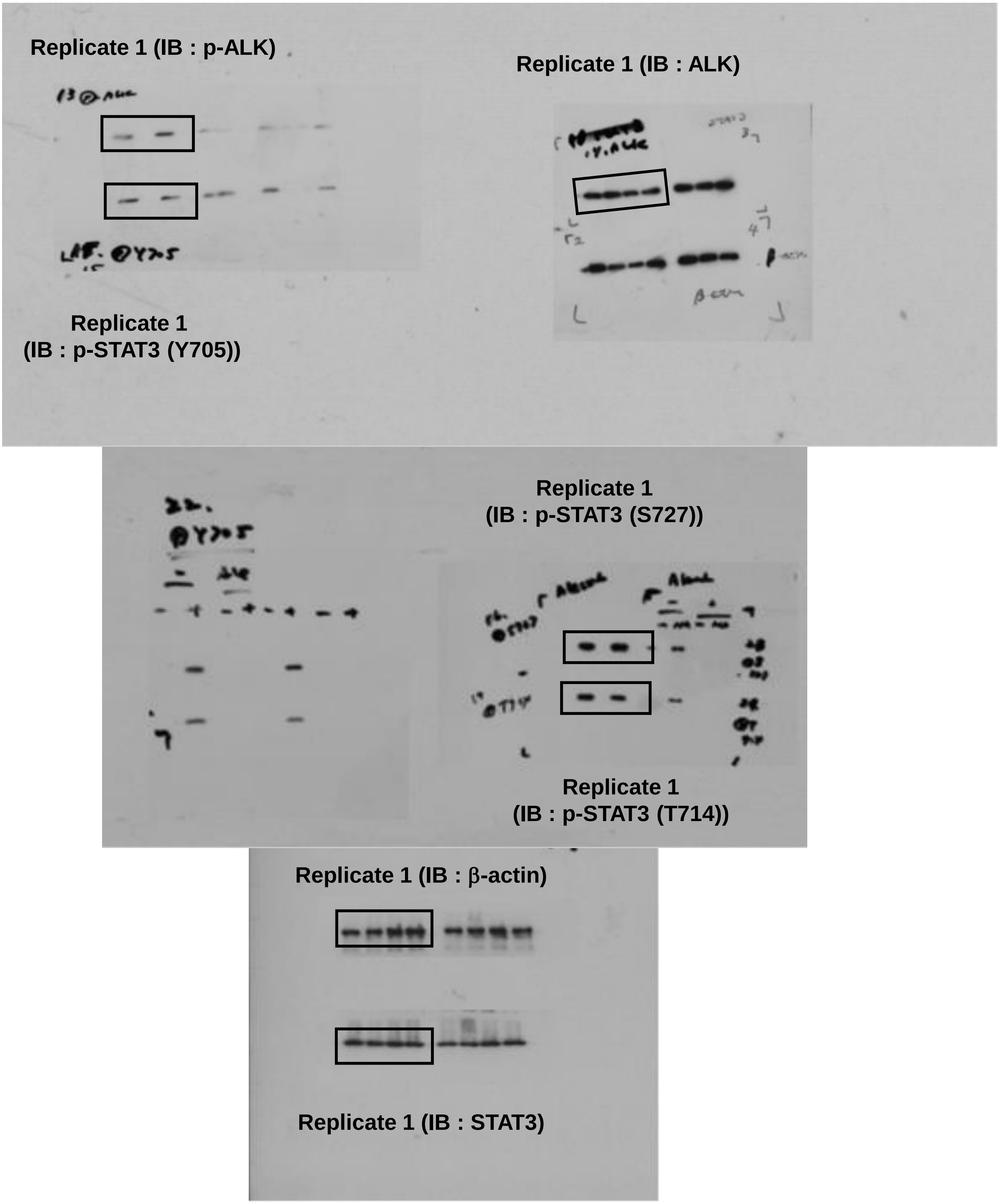

Original blot images, including all replicates used Figure 2B

Fig. 2B

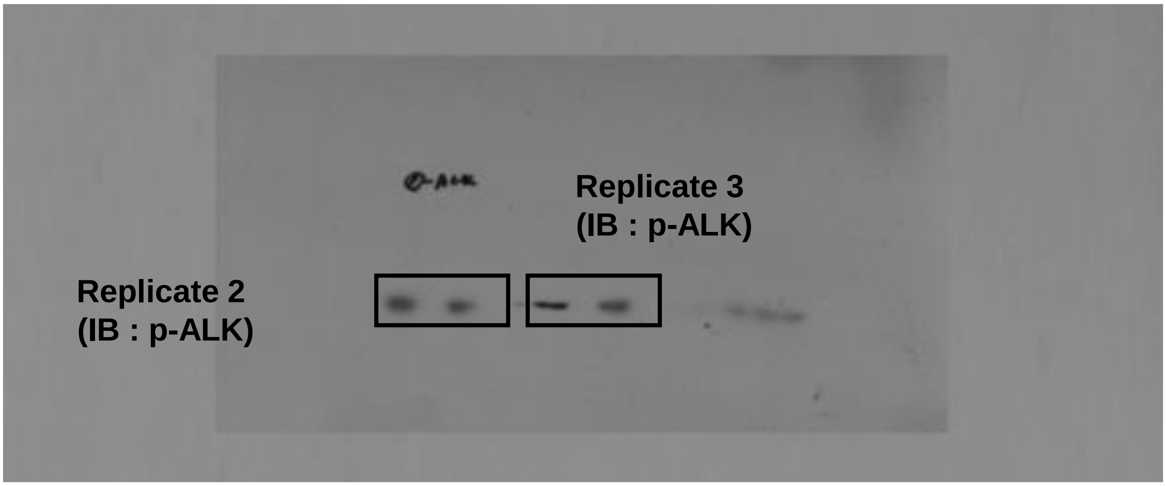

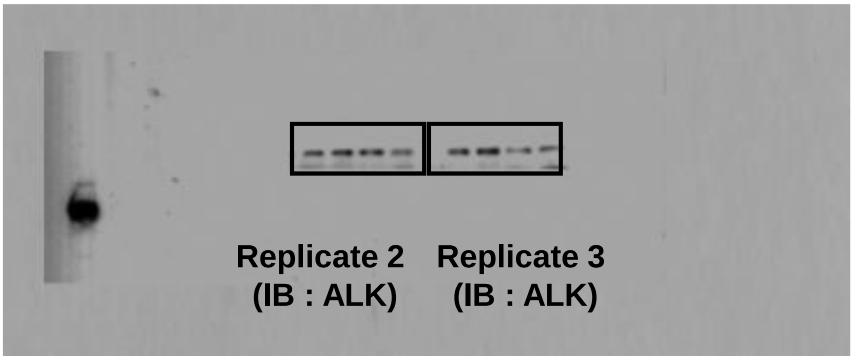

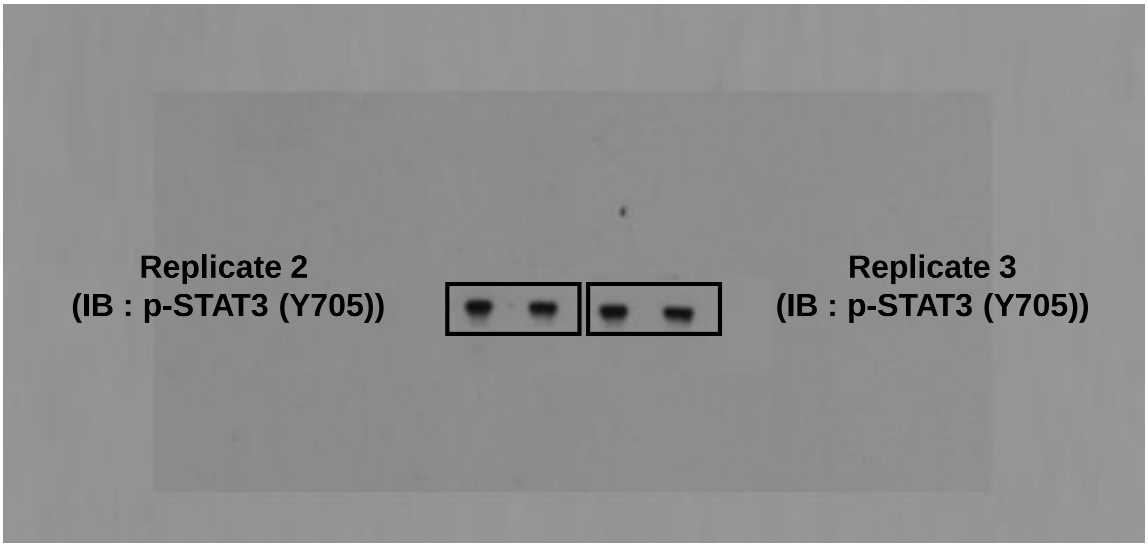

Original blot images, including all replicates used Figure 2B

Fig. 2B

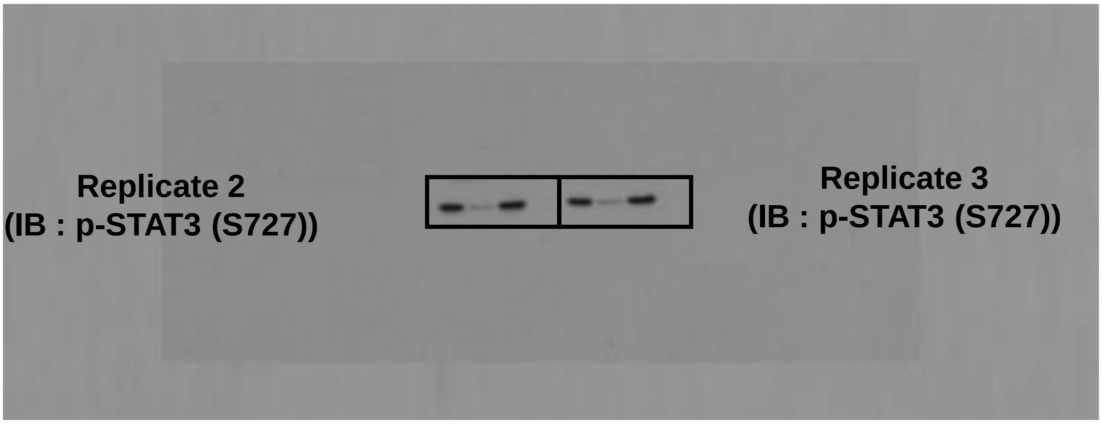

Replicate 2
(IB : p-STAT3 (T714))

Replicate 3
(IB : p-STAT3 (T714))

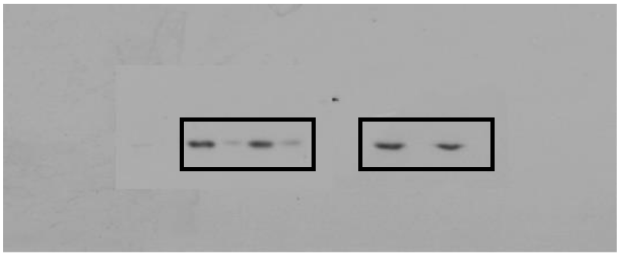

Replicate 2
(IB : STAT3)

Replicate 3
(IB : STAT3)

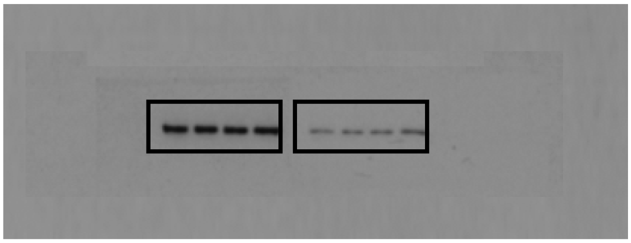

Replicate 2
(IB : β-actin)

Replicate 3
(IB : β-actin)

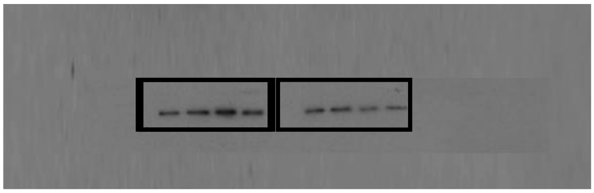

Original blot images, including all replicates used Figure 3A

Fig. 3A

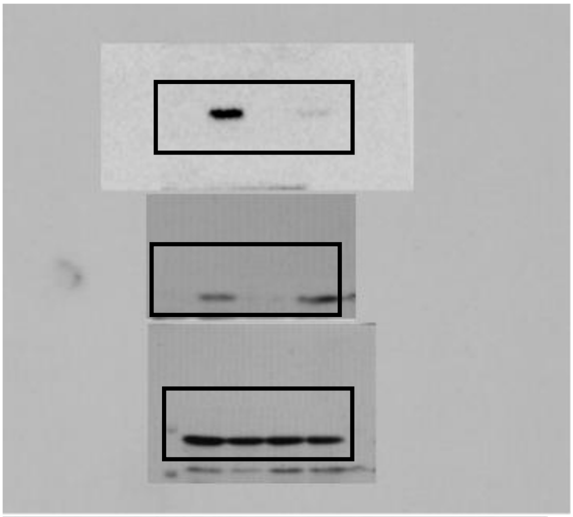

Replicate 1
(IB : p-ALK)

Replicate 1
(IB : ALK)

Replicate 1
(IB : β-actin)

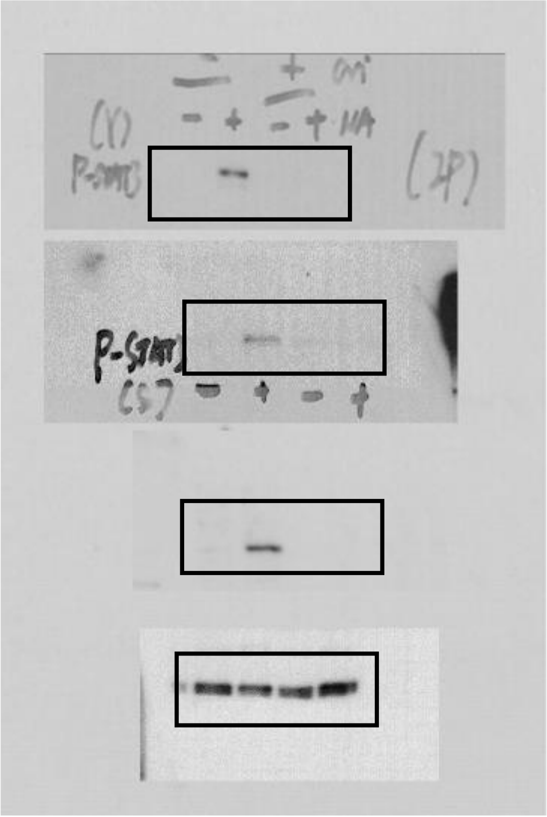

Replicate 1
(IB : p-STAT3 (Y705))

Replicate 1
(IB : p-STAT3 (S727))

Replicate 1
(IB : p-STAT3 (T714))

Replicate 1
(IB : STAT3)

Original blot images, including all replicates used Figure 3A

Fig. 3A

Replicate 2 (IB : p-ALK) Replicate 3 (IB : p-ALK)

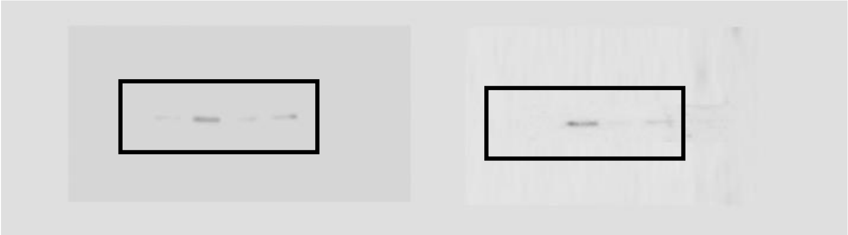

Replicate 2 (IB : ALK) Replicate 3 (IB : ALK)

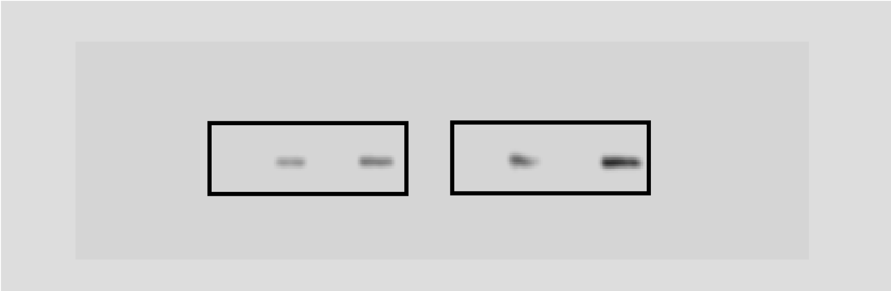

Replicate 2 (IB : β-actin) Replicate 3 (IB : β-actin)

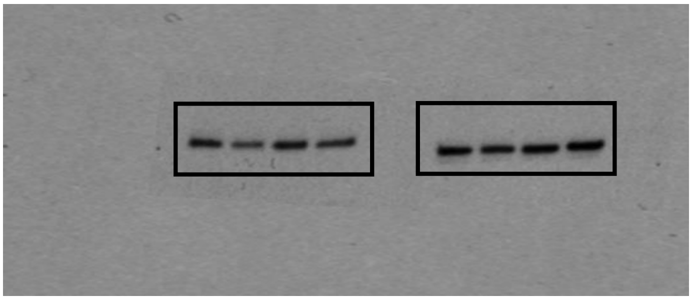

Original blot images, including all replicates used Figure 3A

Fig. 3A

Replicate 2 (IB : p-STAT3 (Y705)) | Replicate 3 (IB : p-STAT3 (Y705))

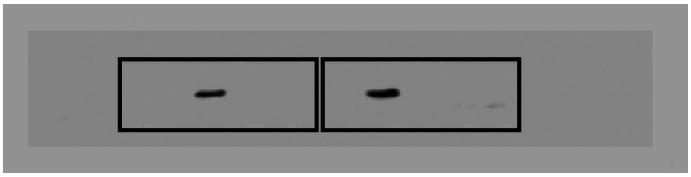

Replicate 2 (IB : p-STAT3 (S727)) | Replicate 3 (IB : p-STAT3 (S727))

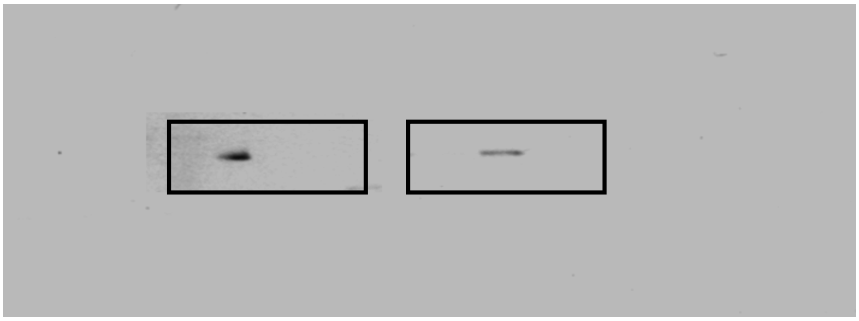

Replicate 2 (IB : p-STAT3 (T714)) | Replicate 3 (IB : p-STAT3 (T714))

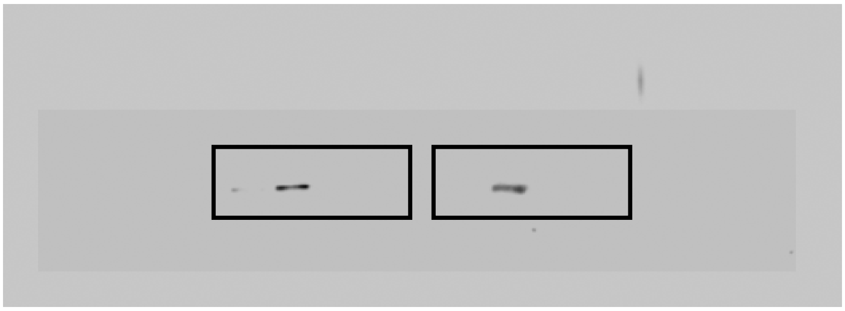

Replicate 2 (IB : STAT3) | Replicate 3 (IB : STAT3)

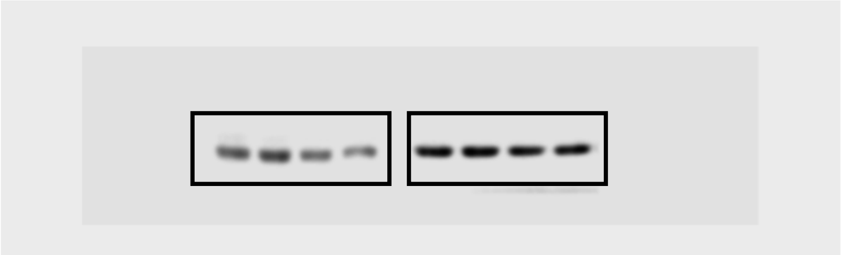

Original blot images, including all replicates used Figure 3B

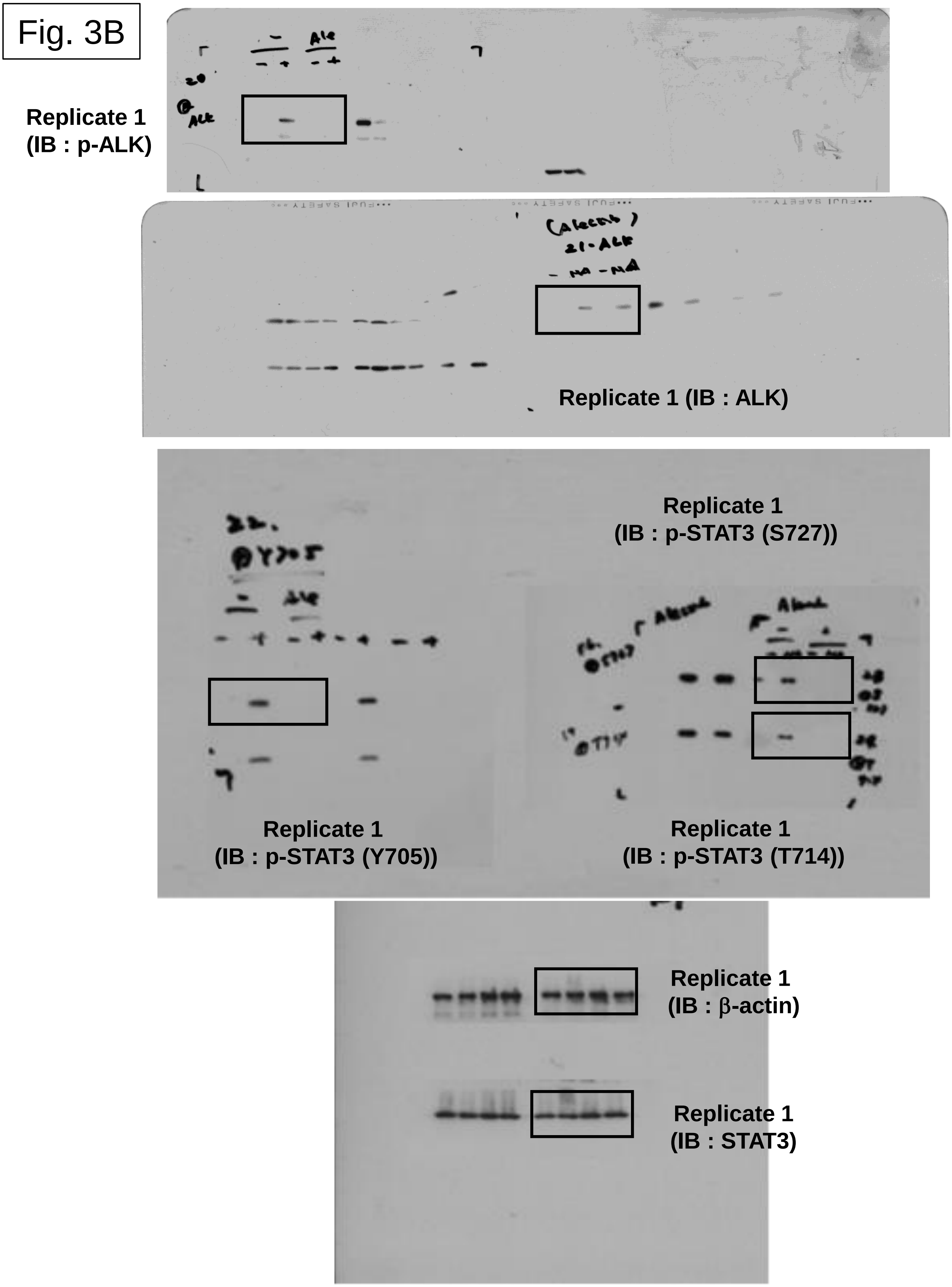

Original blot images, including all replicates used Figure 3B

Fig. 3B

Replicate 2
(IB : p-ALK)

Replicate 3
(IB : p-ALK)

Replicate 2
(IB : ALK)

Replicate 3
(IB : ALK)

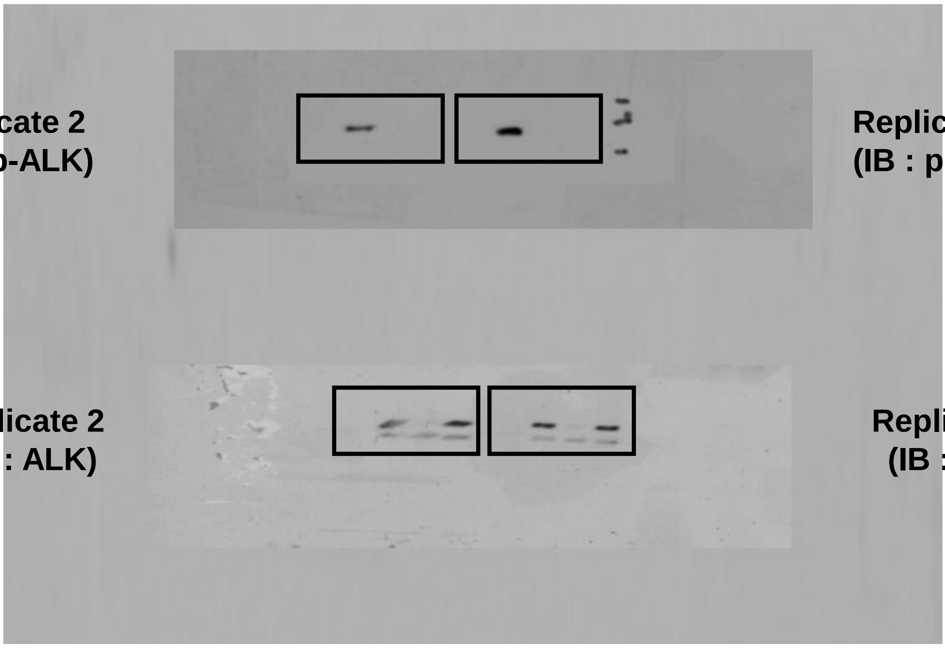

Replicate 2
(IB : p-STAT3 (Y705))

Replicate 3
(IB : p-STAT3 (Y705))

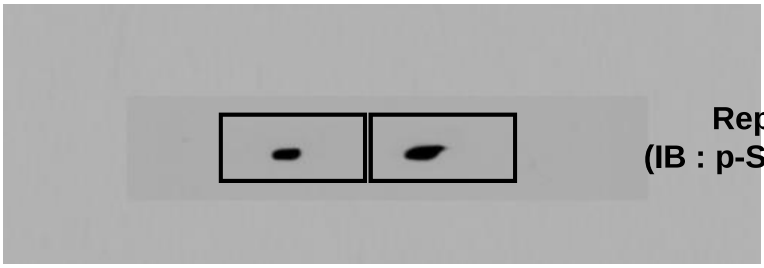

Replicate 2
(IB : p-STAT3 (S727))

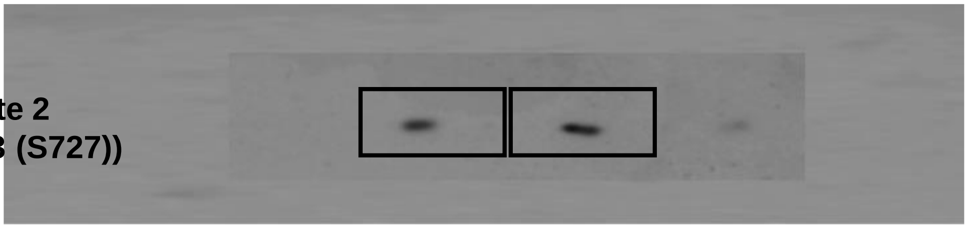

Replicate 2
(IB : p-STAT3 (S727))

Replicate 2
(IB : p-STAT3 (T714))

Replicate 3
(IB : p-STAT3 (T714))

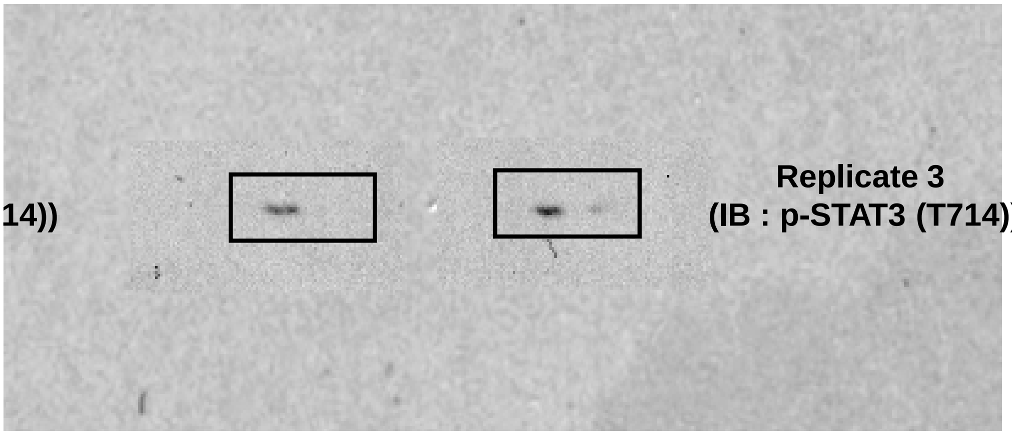

Original blot images, including all replicates used Figure 3B

Fig. 3B

Replicate 2 (IB : STAT3) **Replicate 3 (IB : STAT3)**

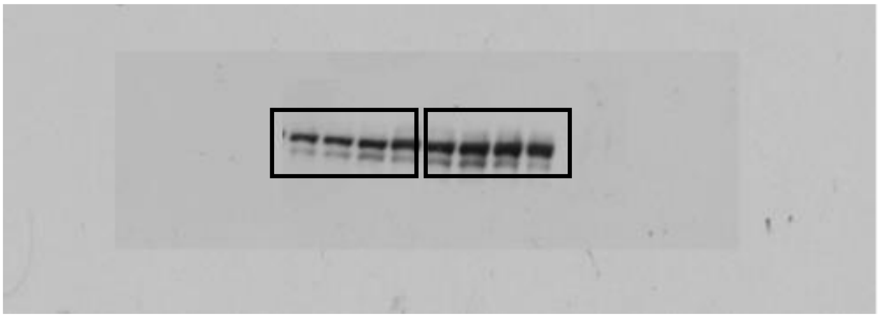

Replicate 2 (IB : β-actin) **Replicate 3 (IB : β-actin)**

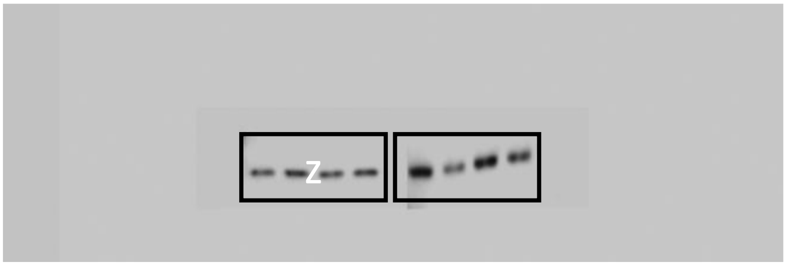

Original blot images, including all replicates used Figure 4

Fig. 4

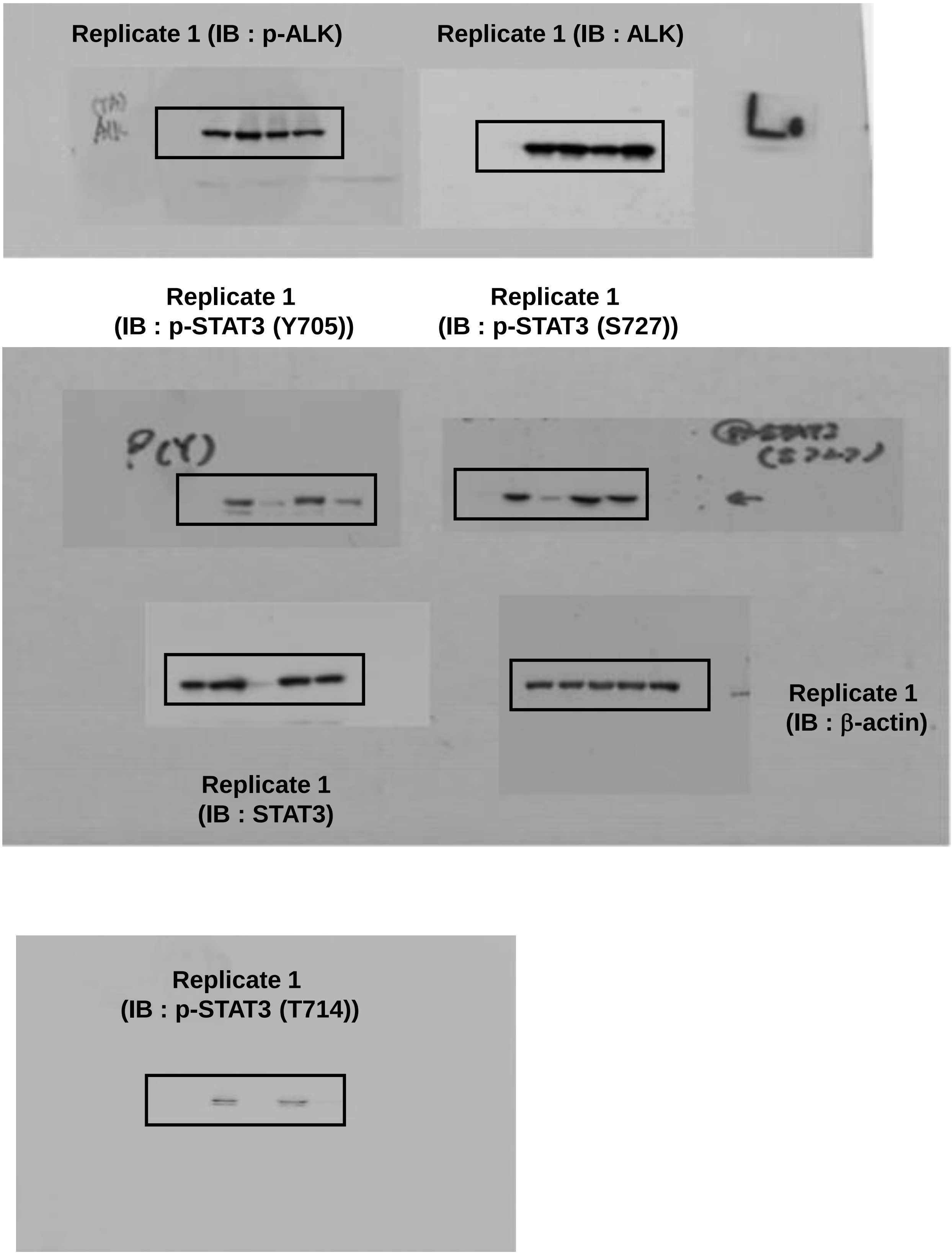

Original blot images, including all replicates used Figure 4

Fig. 4

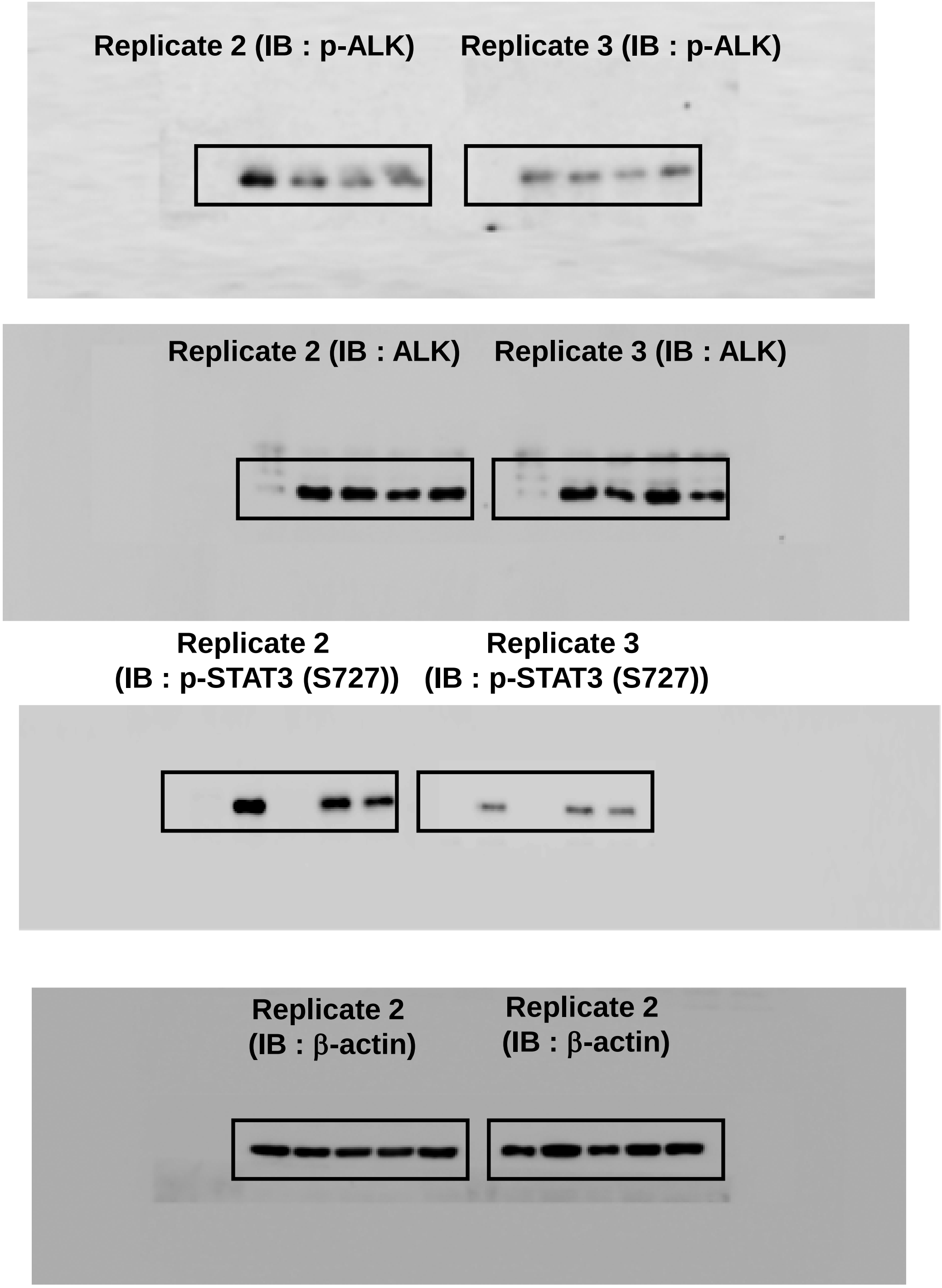

Original blot images, including all replicates used Figure 4

Fig. 4

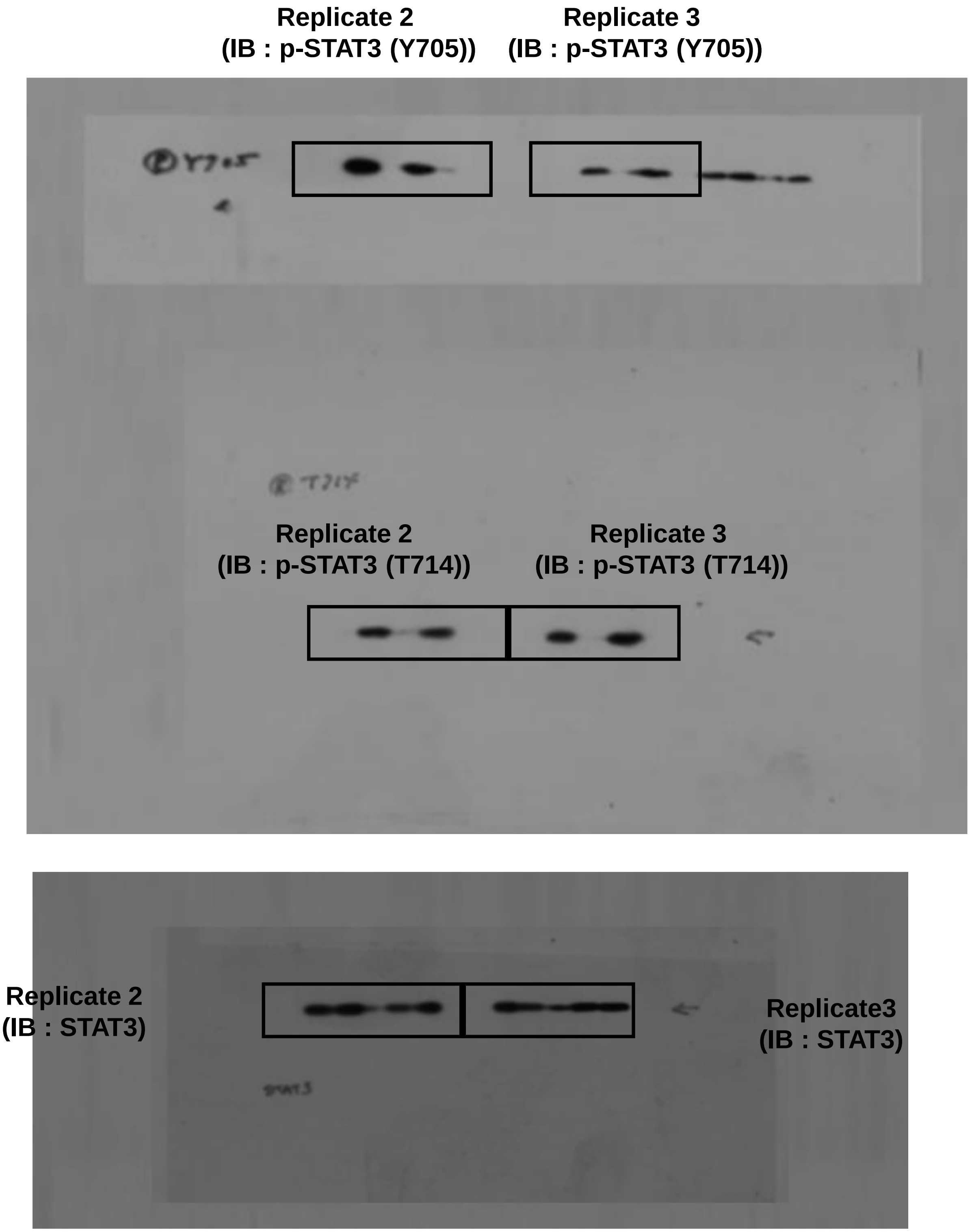

Original blot images, including all replicates used Figure 5A

Replicate 1 (IB : STAT3)

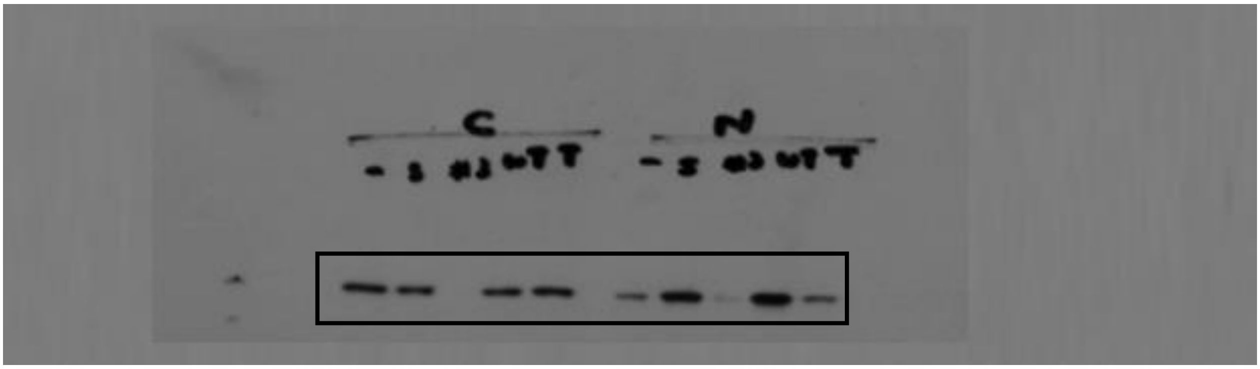

Replicate 1 (IB : MEK1/2)

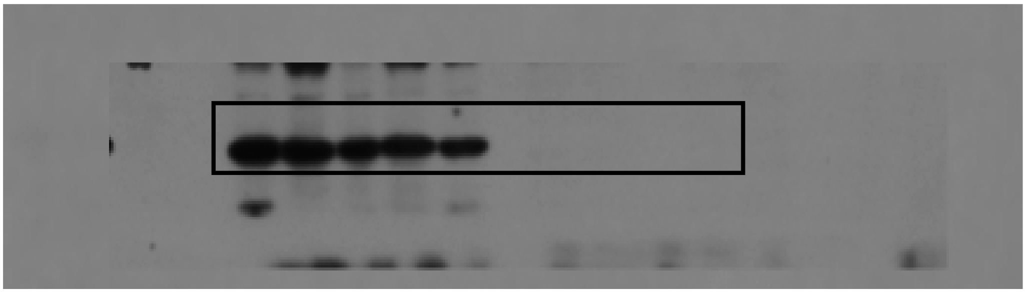

Replicate 1 (IB : Lamin B)

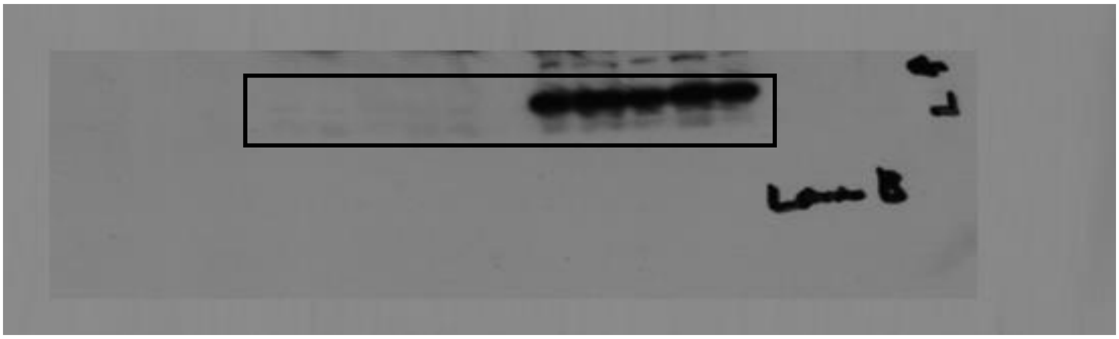

Original blot images, including all replicates used Figure 5A

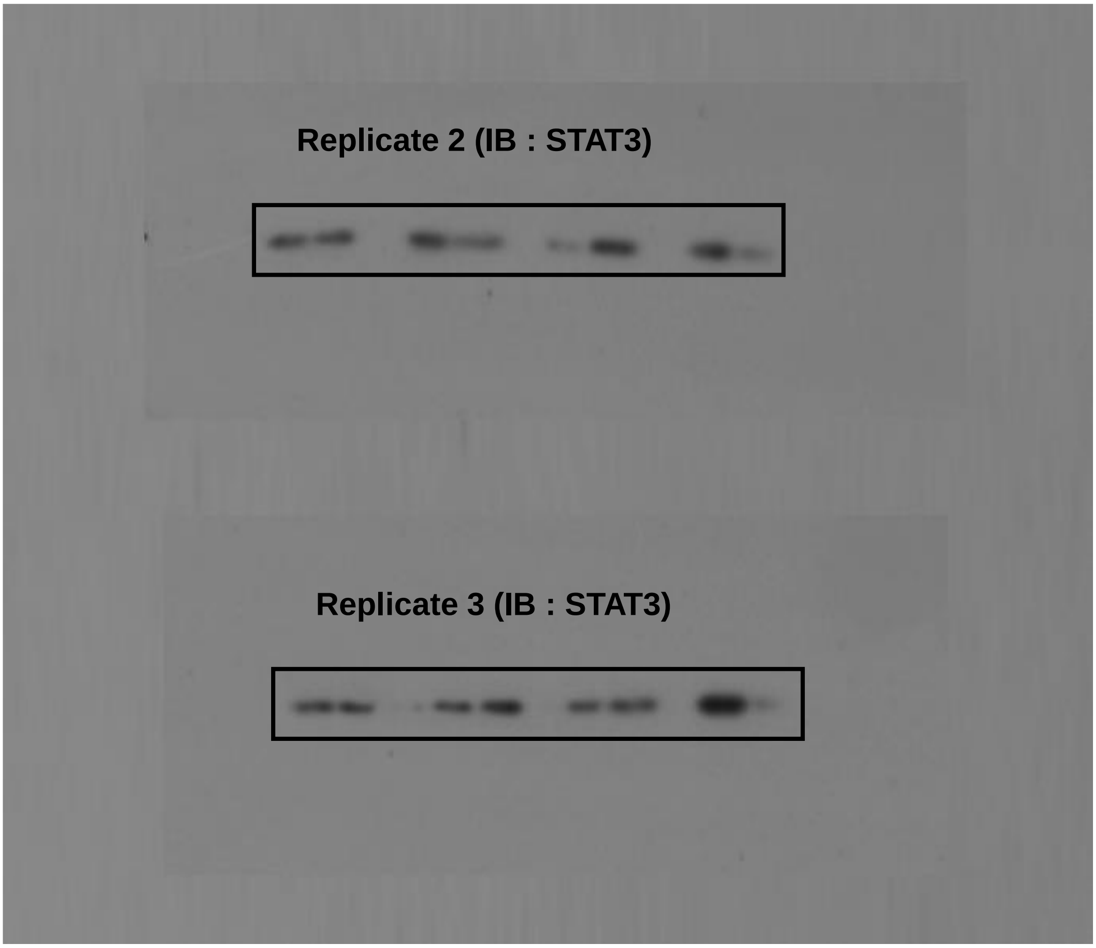

Original blot images, including all replicates used Figure 5A

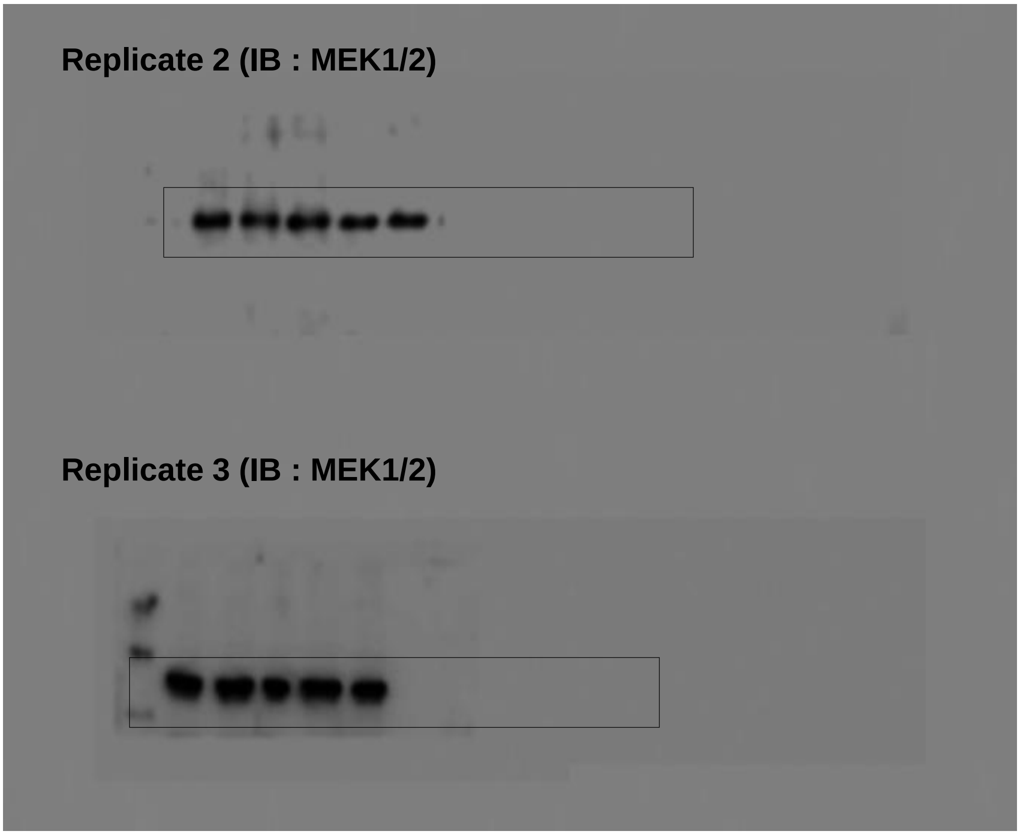

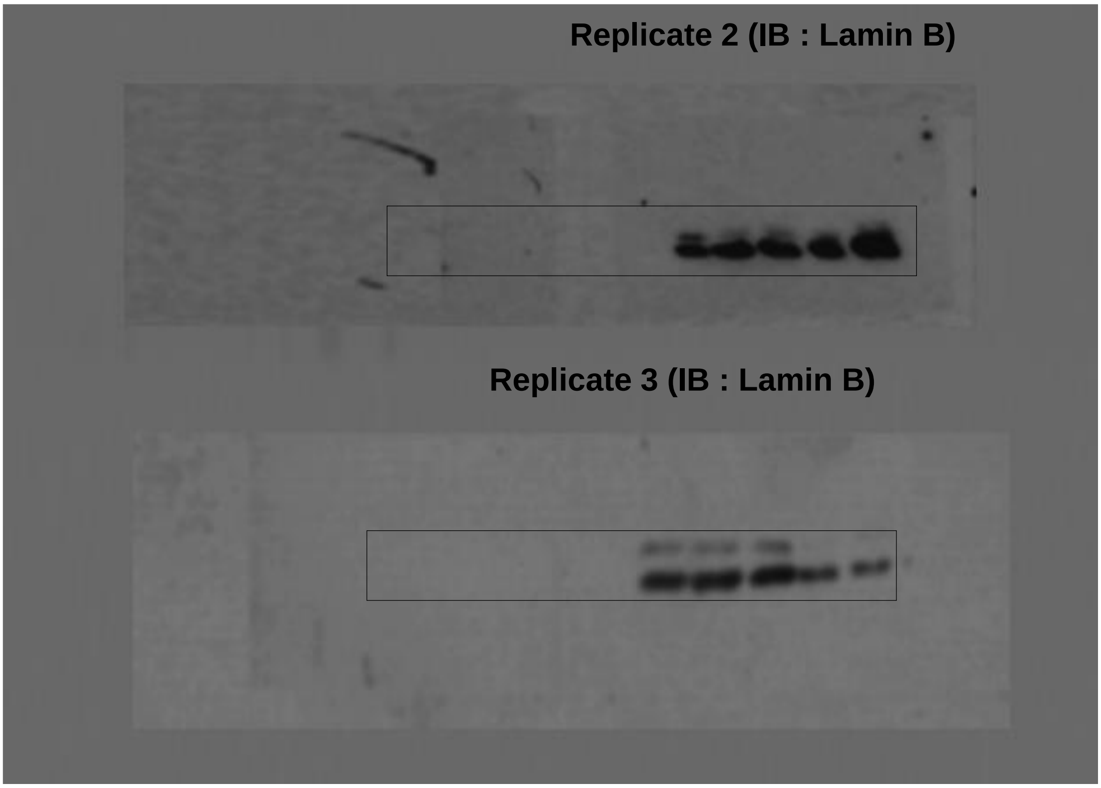

Original blot images, including all replicates used in Supplemental Figure 1

Supplemental Figure 1

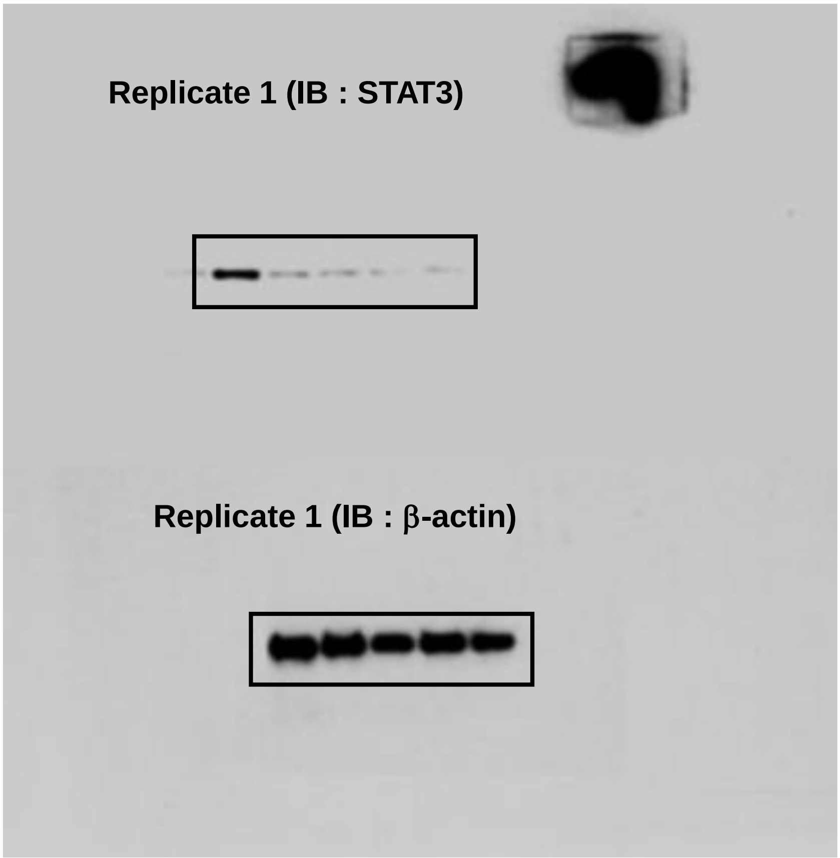

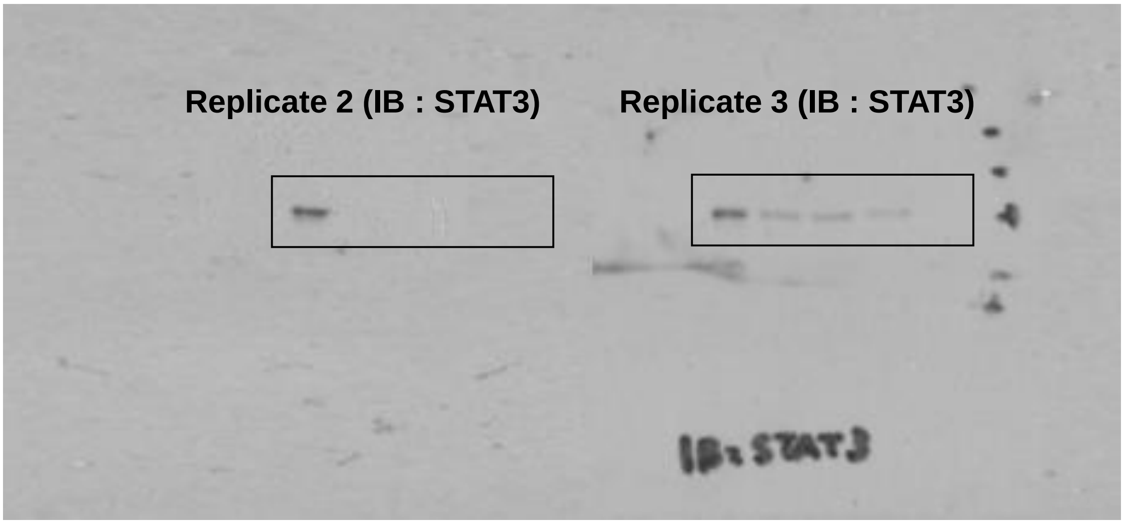

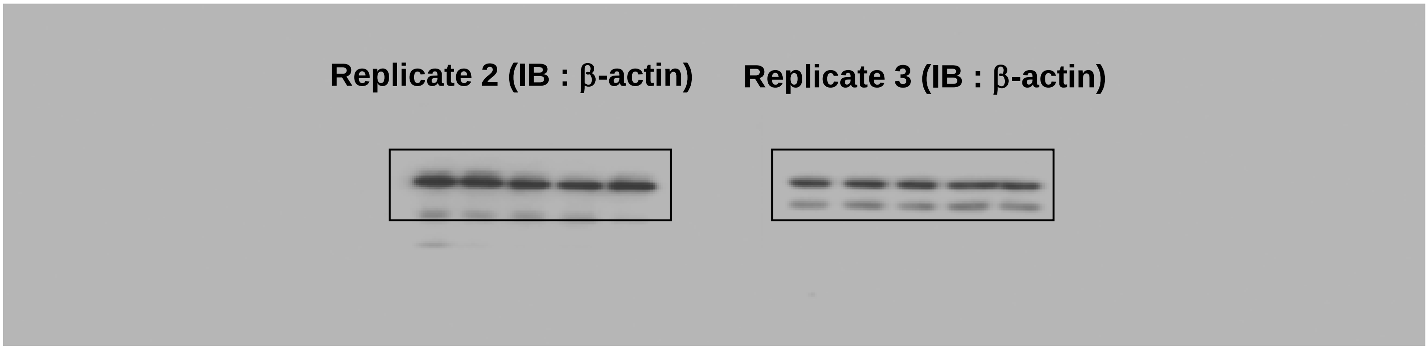

Supplement: Supplementary file 4 — Supplementary Material 4 [file 41598_2026_44867_MOESM4_ESM.pdf]
