# Supplementary material for: A critical role for STAT3 Thr714 phosphorylation in NPM-ALK-driven tumorigenesis
Source: Sci Rep. 2026 Mar 25;16:15005. doi: 10.1038/s41598-026-44867-w (PMC13172448; doi:10.1038/s41598-026-44867-w)
Supplement: Supplementary file 5 — Supplementary Material 5 [file 41598_2026_44867_MOESM5_ESM.docx]

**Supplemental Figure 1. Sustained knockdown of STAT3 in Ba/F3 cells expressing NPM–ALK.**

Ba/F3 cells expressing NPM–ALK were infected with retroviral shRNA targeting STAT3 and selected with puromycin. After removal of puromycin, cells were harvested at the indicated time points (0, 24, 48, 72, and 96 hours). (A) Whole-cell lysates were prepared and subjected to immunoblotting to evaluate STAT3 protein levels. **The membranes were cut prior to antibody incubation,** Quantification of STAT3 expression was performed (n = 3). Statistical significance was determined at ******p < 0.01. **All original blot images, including all replicates, are provided in the Supplementary Information.** (B) Total RNA was extracted and RT-PCR was performed to assess STAT3 mRNA expression. Relative expression levels were calculated using β2-Microglobulin as an internal control (n = 3). Statistical significance was determined at **p < 0.001.
